# Indoor residual spraying for the control of visceral leishmaniasis: A systematic review

**DOI:** 10.1371/journal.pntd.0010391

**Published:** 2022-05-19

**Authors:** Claudia Faber, Carlos Montenegro Quiñonez, Olaf Horstick, Kazi Mizanur Rahman, Silvia Runge-Ranzinger

**Affiliations:** 1 Heidelberg Institute of Global Health, University of Heidelberg, Heidelberg, Germany; 2 Instituto de Investigaciones, Centro Universitario de Zacapa, Universidad de San Carlos de Guatemala, Zacapa, Guatemala; 3 The University of Sydney, University Centre for Rural Health, Lismore, New South Wales, Australia; University of Iowa, UNITED STATES

## Abstract

Indoor Residual Spraying (IRS) is one of the interventions to control the vectors of Visceral Leishmaniasis (VL). Different insecticides are used in affected countries, also in the Regional Initiative for the Elimination of VL in South-East Asia. This systematic review assesses all available studies analysing the effectiveness of IRS on the key vectors of VL. The systematic review followed PRISMA guidelines, with a broad search strategy, applied to seven key databases. Inclusion criteria were studies focusing on 1) Visceral leishmaniasis 2) Indoor Residual Spraying (IRS) or synonyms, and 3) all primary research methods. 21 studies were included, five cluster randomised controlled trials (cRCTs), one randomised controlled trial (RCT), 11 intervention studies, also included were three modelling studies and one survey. 19 out of 21 included studies were published between 2009 and 2020. 18 of the studies were conducted in the context of the Regional Initiative. Effects of IRS on vector populations are positive, confirmed in terms of effectiveness and by the availability of studies. Deltamethrin and alpha-Cypermethrin reduce total sandfly counts, and/or *Phlebotomus argentipes* counts by up to 95% with an effect of a minimum of one month. Prolonged effects are not regularly seen. DDT has been used in India only: whereas in the 1990s a good effect could be measured, this effect waned over time. Two intervention studies, embedded in larger programmes in 2019 and 2020, replaced DDT with alpha-Cypermethrin throughout the study. Combinations of different interventions are not systematically researched, however showing some promising results, for example for the combination of IRS and Temephos. Constant monitoring of insecticide resistancies and quality delivery of IRS are confirmed as key issues for programmes. No human transmission data are available to directly relate an effect of IRS–although modelling studies confirm the effect of IRS on human transmission. Concluding, IRS continues to be an effective intervention for *Phlebotomus argentipes* control. Delivery requires constant monitoring and quality assurance. Further studies need to assess IRS in different geographical areas affected by VL and combinations of interventions.

## Author summary

Visceral leishmaniasis (VL) is a vector borne disease endemic in Southeast Asia (SEA) and present in other regions of the world. VL is transmitted by different species of sandflies and key strategies against VL rely on the control of the vectors. This systematic review assesses the efficacy and community effectiveness of indoor residual spraying (IRS) in controlling sandflies and VL occurrence among humans. After a revision of 8822 initial hits, 21 studies were included in this systematic review.

Effects of IRS on vector populations are positive, Deltamethrin and alpha-Cypermethrin reduce total sandfly counts by up to 95% for at least one month. DDT has been used in India only: whereas in the 1990s a good effect could be measured, this effect waned over time. Combinations of different interventions are not systematically researched, however showing some promising results, for example for the combination of IRS and Temephos. Constant monitoring of insecticide resistancies and quality delivery of IRS are confirmed as key issues for control programmes. No human transmission data are available to directly relate an effect of IRS on human transmission,–although by modelling studies an effect of IRS on human transmission can be demonstrated.

## Introduction

Visceral leishmaniasis (VL) is of particular importance in South-East Asia, with the existent Regional Initiative for the Elimination of VL seeing a steady decline in cases in the participating countries Bangladesh, India and Nepal [1]. Whereas the three countries suffered prior to the Regional Initiative the highest burden of disease [2], the disease burden has reached the threshold for elimination in Bangladesh and Nepal [1]. Also India is very close to it, and this success is thought to be related to the Regional Initiative [3]. The threshold for elimination is defined by an annual VL incidence of less than one per 10,000 people at the subdistrict, district, and block levels in Bangladesh, Nepal and India, respectively [4]. A 2012 estimate showed 200,000 to 400,000 reported VL cases from 79 countries and 20,000 to 40,000 deaths annually in the previous five years [2]. South Asia alone reported 80% of global VL cases at that time, mostly from the countries including Bangladesh, India and Nepal. Data from a more recent year (2015) demonstrated reduction in number of VL cases (23,804) across the globe, accounting South Asia for 39% (9,249) of those cases reportable to WHO [1,5]. In regard to a longer term trend, South Asian countries saw decline of VL cases from 77,000 in 1992 to fewer than 7000 in 2016 [5]. Most of this success in reducing VL incidence has been achieved through a combination strategy of early case detection and treatment, integrated vector management, surveillance with active and passive case detection, including social mobilisation and implementation research [6]. The Regional Initiative has been supported by the World Health Organizations’ Special Programme for Research and Training in Tropical Diseases (WHO/TDR), with a focus on scientific support [7].

As for vector control of VL, there are several interventions documented in a meta-review of existing systematic reviews for vector control of Cutaneous Leishmaniasis (CL) and VL [8]. Of the existing interventions, the most applied are Indoor Residual Spraying (IRS) and the use of Insecticide Treated Nets (ITNs). Both are applied for both forms of leishmaniasis, CL and VL. However, the use of vector control methods, and its efficacy and community effectiveness are continuously under discussion, particularly with changing resistance patterns and with different levels of transmission [9]. Fitzpatrick (2017) postulated that post-elimination of VL the major challenge is to sustain elimination and to invest in remaining areas of scientific uncertainty [10]. The questions arise, what is the role of vector control for the control of VL, in the context of the changes described, and is existing evidence good enough for public health recommendations?

In this context, this systematic review addresses what is known about the effectiveness of IRS for the control of VL, in the community. A similar systematic review for ITN is in the process of publication. Following the PRISMA statement [11]; the key objective is to systematically review all available evidence for the control of the key vectors of VL through IRS, with a broad search, allowing for all study types, but not including opinion statements and reports.

## Methods

### Search strategy, databases and search terms

In response to a gap analysis undertaken in the above-mentioned meta-review of vector control interventions for the control of CL and VL [8], this systematic review has been undertaken. It focuses therefore on the role of IRS for the control of VL. Key difference to previously published work is the inclusion of studies with randomisation, but also those without randomisation, considering that a lot of studies analysing vector control methods have been undertaken without randomisation processes and in the context of ongoing control programmes.

As a first step a study protocol was established. All searches have been carried out until 17 of October 2020, using the following databases: Google Scholar (considering the database is sorted by relevance, we screened the first 200 hits only, after establishing that for most of our searches no relevant hits were found after screening the first 100 hits [12]), Lilacs, PubMed, ScienceDirect, WHOlis, WHOiris and PAHOiris.

The searches were performed without restriction to language, publication year or region of publication. Searches were performed in English with a focus on primary research articles with no restriction to a specific study design.

1. Disease: “Visceral Leishmaniasis” (MeSH term, where applicable, major topic where applicable)
2. Intervention: “IRS” or “Indoor Residual Spraying” and its potential variations (MeSH term, where applicable)
3. Vectors for transmission of visceral leishmaniasis: “Phlebotomus” or “Lutzomyia”

Inclusion criteria were all studies focusing on 1) Visceral leishmaniasis, 2) Indoor Residual Spraying (IRS) or synonyms, and 3) all primary research methods were included.

Exclusion criteria were conference or opinion articles, editorials, or any articles without clear primary research methodology. Furthermore, the reference lists of all included articles were manually searched for additional articles. As for grey literature, guidelines, not older than 5 years, for VL were screened, including reference lists.

All searches performed have been documented, including the selection process. Two data extractors (CAMQ and CB) independently screened titles and abstracts and applied inclusion and exclusion criteria. In case of disagreement a third researcher (SRR) was involved to reach consensus.

The included studies have been categorised for further analysis into the following categories: 1) Studies, or study arms, assessing effectiveness of IRS for the control of VL with 1.1) cRCTs, 1.2) RCTs, 1.3) Intervention studies and 2) All other study types contributing to formal effectiveness assessments of IRS with 2.1) Modelling studies with estimated effectiveness and 2.2) Surveys with perceived effectiveness and/or establishment of risk factors of VL. We also aimed to analyse according to geographical regions, due to the different vectors involved in the context of VL.

### Quality assessment

Quality assessment has been performed using the CONSORT checklist (http://www.consort-statement.org/) for a) RCTs and cRCTs applying all 25 items of the checklist. For b) other studies, CONSORT has been used, excluding the criteria for randomisation and therefore applying 19 items of the checklist. Depending on the study design this denominator was set as 100% respectively. Studies have not been excluded following the quality assessment, but analysis and reporting reflected the quality assessment.

### Data extraction and analysis

Data have been extracted in a predefined data extraction matrix and analysed by author, year of publication, country/geographical region, study type, methods used, sample size, follow up period, study arms and interventions included, insecticides used, different vectors, entomological outcome indicators, human outcome indicators, reported conclusions, limitations, and quality assessment scores. Evidence tables and results summary tables were developed for better presentation of data and analysis.

## Results

### Descriptive results

#### Results of searches

**Fig 1:**
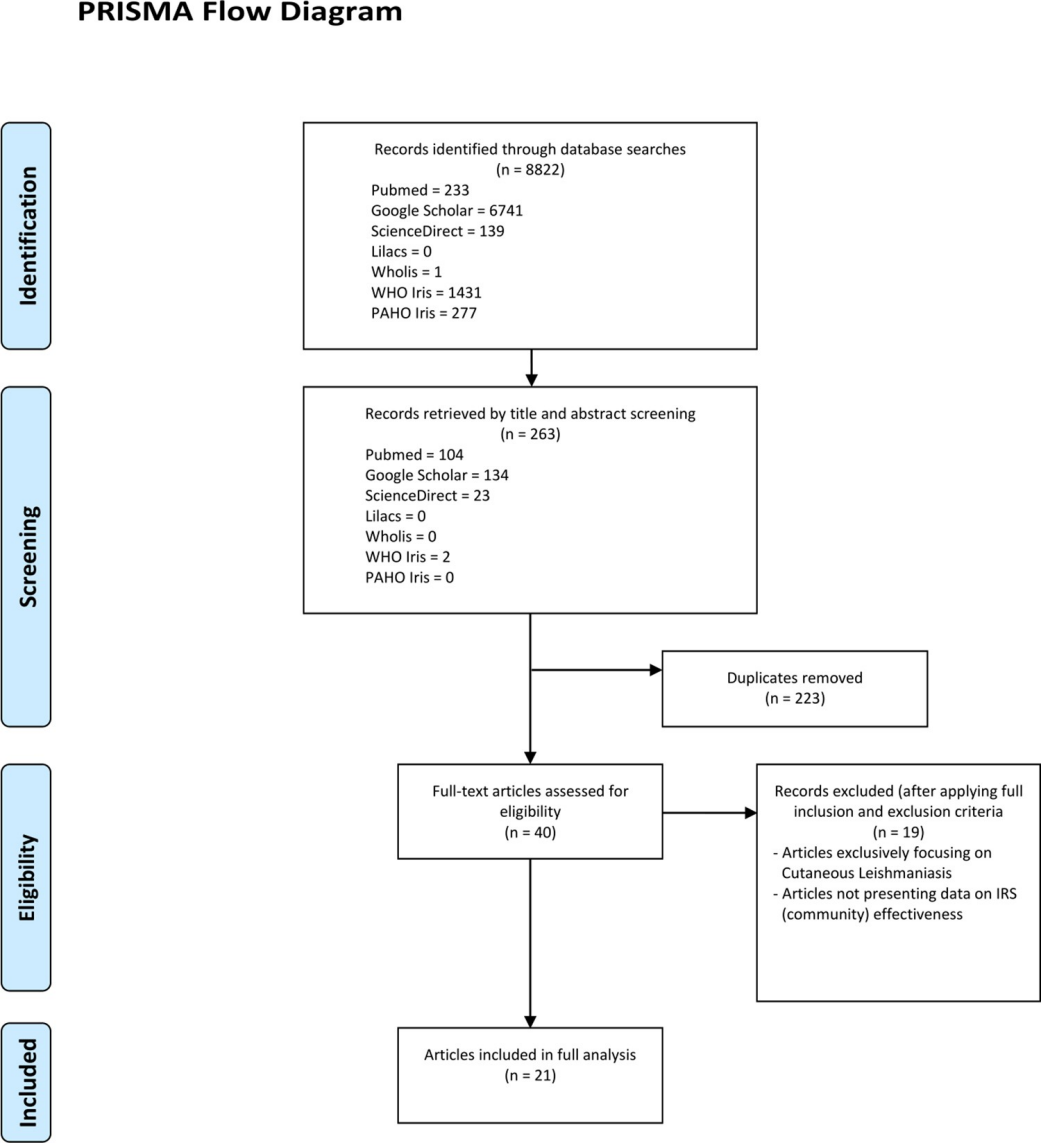
PRISMA flowchart for searches and inclusion of articles for Systematic Review on the use of indoor residual spraying for visceral leishmaniasis control [13].

8822 initial hits were identified on the seven databases (Fig 1: Prisma flowchart). Title and abstract screening focused on 263 potentially relevant articles, with 223 duplicates. With the large number of duplicates, most studies were identified on PubMed and equally on Google Scholar. 40 articles were fully assessed. After application of full inclusion and exclusion criteria, 19 articles were further excluded, mostly since the focus was not VL and its vectors. 21 studies were fully included, with 17 studies assessing effectiveness of IRS (five cRCTs, one RCT, 10 intervention studies in Asia, one intervention study in Latin America) and further three modelling studies and one survey. The last two groups were included, although not directly assessing effectiveness, but informing about the estimated or perceived effects of IRS.

#### Description of included studies

*Time and geographical clustering of included studies*. Of the 17 included effectiveness studies and four further studies, the vast majority (20/21) were conducted in the geographical context of the Regional Initiative, or even embedded in the Regional Initiative, with only one study [14] from Brazil (Tables 1 and S1). Furthermore, of the five cRCTs, four focused on Bangladesh [4,15,16] of which one was conducted in Bangladesh, India and Nepal [17] and one further study in Nepal only [18]. The only RCT was equally conducted in Nepal [19]. As for the 10 intervention studies in the geographical area of the Regional Initiative, most studies were implemented in India, only Chowdhury (2018) is from Bangladesh and Chowdhury (2011/2) from India and Nepal [20,21].

**Table 1:** Summary IRS result table.

Author, year, country	Study type/ Methodology	Sample size/ Follow-up	Study arms /Interventions in detail	Species of Vector/ Species of parasite	Results: Human indicators	Results: Entomological indicators Intervention	Results: Entomological indicators Control	Results: Entomological indicators Percentage reductions	Quality assessment scores by the authors
**cRCTs**									
Huda 2019 [4]	cRCT	Bangladesh Fulbaria and Trishal Mymensingh district Intervention: Fulbaria Control: Trishal 12 HH in three clusters each	Sand fly density/CDC light traps Baseline prior to IRS, At 1 month, 3 months, 6 months, 9 months and 12 months post-IRS Deltamethrin, and deployment of larvicide (Temephos 50 EC, 5 mL/10 L)	*Phlebotomus argentipes*	18 house to house searches with 539 HH and 2505 attendants have not identified fever cases of more than 2 weeks, or PKDL like skin lesions	Total *P*. *argentipes* captured (Here: female only, SD) Baseline 0.67 (0.93) 1 month 0.81 (1.12) 3 months 1.03 (1.13) 6 months 0.64 (0.83) 9 months 0.69 (1.04) 12 months 0.36 (0.83)	Total *P*. *argentipes* captured (Here: female only, SD) Baseline 0.44 (0.73) 1 month 2.58 (4.54) 3 months 5.39 (7.87) 6 months 1.81 (2.91) 9 months 1.14 (1.46) 12 months 1.17 (1.78)	Here effect on count - 2.00 (-0.77, -3.23) - 4.59 (-2.24, -6.93) - 1.40 (-0.61, -2.16) - 0.68 (-0.46, -0.87) - 1.04 (-0.63, -1.42) The reduction in the incidence rate of female *P*. *argentipes* sandfly count was 74% at 12-month follow-up for IRS/Temephos	17/25
Chowdhury 2017 [15]	cRCT	Bangladesh Fulbaria, Mymensingh district 5 HH in 10 sections with 6 clusters each	Sand fly density/CDC light traps Baseline prior to IRS Follow up: 2, 4, 5, 7, 11, 14, 15, 18 and 22 months post-IRS Alpha Cypermethrin 5 WP	*Phlebotomus argentipes*		Total *P*. *argentipes* captured (Here: per HH) Baseline 11.53 (10.35–12.81) 2 month 0.17 (0.05–0.39) 4 months 0.37 (0.18–0.66) 5 months 2.17 (1.67–2.76) 7 months 3.77 (3.1–4.53) 11 months 0.33 (0.16–0.61) 14 months 0.1 (0.02–0.29) 15 months 0.83 (0.54–1.23) 18 months Not done 22 months Not done	Total *P*. *argentipes* captured (Here:per HH) Baseline 10.57 (9.44–11.8) 2 month 0.73 (0.46–1.11) 4 months 3.13 (2.53–3.83) 5 months 12.1 (10.89–13.41) 7 months 10.83 (9.69–12.08) 11 months 3.8 (3.13–4.56) 14 months 0.83 (0.54–1.23) 15 months 4.87 (4.11–5.72) 18 months 20.27 (18.69–21.94) 22 months 9.83 (8.74–11.02)	The reduction of sand fly vector densities was 14% to 80% at the different points of measurement and the rate ratio (RR) of *P*. *argentipes* sand fly counts after and before the intervention was 0.86 and 0.20 up to a 15 months period.	18/25
Chowdhury 2011/1 [16]	cRCT	Bangladesh Fulbaria, Mymensingh district. 4 villages were randomly selected from the 20 villages, each village was divided into 6 geographic clusters (24 clusters). 120 HH were selected for vector collection.	Sand fly density/CDC light traps Baseline prior to IRS, At 1 month, 2 months, 4 months, 5 months and 12 months post-IRS Deltamethrin	*Phlebotomus argentipes*		Total *P*. *argentipes* captured Baseline 595 1 month - 2 months 8 4 months 261 5 months 129 12 months 60	Total *P*. *argentipes* captured Baseline 632 1 month - 2 months 51 4 months 1240 5 months 652 12 months 409	(Here: Change in Rate Ratios) 0.99 (0.70–1.41) - 0.15 (0.05–0.46) 0.24 (0.17–0.35) 0.21 (0.14–0.32) 1.57 (1.09–2.25)	16/25
Das 2010 [18]	cRCT	Nepal Sunsari and Morang 24 clusters with 1335 HHs and 6955 habitants. November 2006 to April 2007	CDC light traps and standard mouth suction aspiration Baseline 2 weeks prior to IRS 2 weeks, 4 weeks and 5 months post-IRS Alpha-cypermethrin	*Phlebotomus argentipes*, *P*. *papatasi*, *Sergentomyia spp*.		*P*. *argentipes* captured per trap (and CI) Baseline 11.0 (6.0–20.3) 2–4 survey mean 0.6 (0.3–1.3) *P*. *argentipes* captured through aspiration (and CI) Baseline 1.3 (0.7–2.4) 2–4 survey mean 0.0 (0.0–0.1)	*P*. *argentipes* captured per trap (and CI) Baseline 5.7 (3.6–8.9) 2–4 survey mean 2.3 (1.1–4.9) *P*. *argentipes* captured through aspiration (and CI) Baseline 1.8 (1.1–2.9) 2–4 survey mean 0.8 (0.6–1.0)	P = 0.070 0.009 0.391 <0.001	11/25
Joshi 2009 [17]	cRCT	Bangladesh, India and Nepal 120 HH in 24 clusters, with 6 clusters for IRS	Sand fly density/CDC light traps (2 nights) Baseline prior to IRS, At 5 months in India and Nepal and 6 months in Bangladesh post-IRS Deltamethrin in Bangladesh DDT in India Alpha cypermethrin in Nepal	*Phlebotomus argentipes*		Total sandflies captured per trap (and CI 95%)) Baseline 12.32 (9.54–12.32) 5 months 6.14 (4.00–10.47)	Total sandflies captured per trap (and CI 95%)) Baseline 19.41 (6.97–12.71) 5 months 12.15 (8.68–17.00) p value in IRS versus control: baseline 0.184, 5 months 0.035	Reduction in counts 72.4%, with variation between 52–124% between sites Nepal site 1 -10.7 (-26.7, 5.3) 53% Nepal site 2 -8.5 (-16.0, -0.9) 52% Bangladesh -10.3 (-13.8, -6.8) 94% India -7.1 (-9.5, -4.7) 124%	16/25
**RCT**									
Banjara 2019 [19]	RCT (for the vector control part of the study)	Nepal, Saptari district Intervention 264 HH Control 92 HH	Sand fly density/CDC light traps Baseline prior to IRS Follow-up after 1, 3, 9 and 12 months post-IRS Deltamethrin	*Phlebotomus* *argentipes*		Total sandflies captured per HH/night Baseline 2.21 1 month 2.75 3 months 3.36 9 months 3.83 12 months 23.67	Total sandflies captured per HH/night Baseline 6.29 1 month 31.96 3 months 2.42 9 months 1.62 12 months 6.46	Change and 95%CI - 25.13 (-49.23 — –1.02) 5.02 (2.17–7.86) 5.82 (3.11–8.52) 21.29 (17.7–24.87)	15/25
**Intervention studies**									
**Asia**									
Kumar 2020 [3]	Intervention study	India Vaishali district of Bihar For entomological assessment, one intervention village with 2 HH from each of 3 HH types, same in control village (non endemic, not in IRS programme()	CDC light trap Two rounds of IRS were performed annually using 166 IRS squads. In 2015, the first round of IRS was performed using DDT (WP 50% at a dosage of 1 g/m2). In the second round, DDT was used for the first 15 days then SP (alphacypermethrin 5% at 25 mg/m2).	*Phlebotomus argentipes*	All 16 blocks of the Vaishali district achieved the VL elimination target in 2016. VL cases were reduced from 664 in 2014 to 163 in 2016 and endemic villages from 282 in 2014 to 142 in 2016. The case reduction rate was increased from 22.6% in 2014 to 58.8% in 2016. On average, 74 VL infected villages became Kala-azar free each year from 2015 to 2016.	Total *P*. *argentipes* captured (per trap after each round of IRS) Baseline 2.0 (1–17) 3.7 (1–19) 3.4 (1–2016) 3.2 (1–12) 3.3 (1–14) 15 days 1.4 (1–6) 2.3 (1–6) 0 (0–0) 0 (0–0) 0 (0–0) 1 month 1.7 (1–8) 3.4 (1–10) 0.6 (0–2) 0.2 (0–1) 0.5 (0–2) 3 months 2.5 (1–8) 4.7 (1–7) 2.3 (0–2) 1.7 (0–2) 1.8 (0–2)	Total *P*. *argentipes* captured (per trap after each round of IRS) Baseline 2.3 (1–9) 3.3 (1–13) 2.9 (1–14) 3.5 (2–17) 3.2 (2–13) 15 days 2.6 (1–8) 3.4 (2–14) 2.7 (2–18) 3.2 (2–14) 2.9 (1–10) 1 month 2.1 (1–9) 3.6 (1–12) 2.9 (1–8) 3.1 (1–12) 3.3 (1–16) 3 months 2.5 (1–7) 3.5 (1–14) 3.0 (1–10) 3.4 (1–11) 2.8 (2–12)	Percentage change 15 days -46.9 -40.1 -94.6 -90.8 -91.5 1 month -4.6 -17.2 -84.2 -81.5 -85.9 3 months 16.5 19.7 -38.6 -43.9 -33.2 A significant difference was observed in *P*. *argentipes* densities collected in SP sprayed and unsprayed villages during the 15 days, 1 month, and 3 months post-IRS collections (t = 4.6; P <0.01). This difference was observed in the 15 days post-IRS collections (t = 3.2; P <0.05) for DDT-IRS periods. No significant difference was observed in the 1-month and 3-month post-IRS collections (t = <1.7; P = 0.12). At the 15 days post-IRS (SP round), the sand fly RR ranged between 91.5 and 94.6%. A minimum of 81.5% (maximum 85.9%) of the sand fly RR was maintained in SP-sprayed villages for up to 1 month post-IRS. At 3 months post-IRS, the RR ranged from 33.2% to 43.9%. While in the DDT-IRS round in the intervention villages, the sand fly RR at the 15 days post-IRS ranged from 40.1% to 46.9%, and at the 1 month post-IRS, the RR was recorded between 4.6% and 17.2%.	13.5/18
Mandal 2019 [33]	Intervention study	India Vaishali district Intervention 12 HH Control 6 HH	Sand fly density/CDC light traps Baseline 2 weeks prior to IRS, At 2 weeks, 1 month and 3 months post-IRS First round of IRS: DDT (50%) Second round of IRS synthetic pyrethroid (5%)	*Phlebotomus argentipes*		Total *P*. *argentipes* captured First round of IRS Baseline 14.2 (1–28) 2 weeks 6.2 (5–14) 1 month 6.5 (1–14) 3 months 9.5 (1–16) Total *P*. *argentipes* captured Second round of IRS Baseline 10.5 (1–21) 2 weeks 0 (0–0) 1 month 2.3 (2–5) 3 months 8.5 (3–15)	Total *P*. *argentipes* captured Baseline 12.3 (2–14) 2 weeks 9.2 (8–20) 1 month 8.3 (1–18) 3 months 9.7 (9–20) Total *P*. *argentipes* captured Baseline 7.8 (1–17) 2 weeks 6.8 (3–12) 1 month 6.7 (5–13) 3 months 11.7 (1–22)	Percentage reduction - 34.1 - 25.9 - 14.1 - 90.5 - 66.7 - 55.6	15.5/18
Poché 2018 [32]	Intervention study	India Study site: 24 villages in two Bihar districts: Saran and Muzaffarpur (India)	Sand fly density/CDC light traps No baseline Biweekly collection over 47 weeks in 12 villages	*Phlebotomus argentipes*		Total *P*. *argentipes* collection Muzaffarpur 26388 Saran 11952	Data of untreated villages not presented The results of this study 1) provide ecological data regarding *P*. *argentipes* monthly relative abundance, spatial distribution, and host preference comparable to previous research; and 2) suggest relative *P*. *argentipes* abundance within IRS-treated and untreated villages do not differ significantly		13.5/18
Chowdhury 2018 [20]	Intervention study	Bangladesh Mymensingh district Intervention 36 HH in 8 districts (upazilas) Control 36 HH in 8 districts	Sand fly density/CDC light traps Baseline 2 weeks prior to IRS, At one and 5 months post-IRS Deltamethrin 5 WP	*Phlebotomus argentipes*		Total *P*. *argentipes* captured Baseline 930 1 month 1021 5 months 761 *P*. *argentipes* captured per trap (and SD) Baseline 4.36 ± 5.73 (2.42±6.30) 1 month 5.43 ± 6.11 (3.36±7.50) 5 months 5.83 ± 5.79 (3.87±7.79)	Total *P*. *argentipes* captured Baseline 460 1 month 466 5 months 494 *P*. *argentipes* captured per trap (and SD) Baseline 8.71 ± 11.37 (4.86±12.55) 1 month 10.76 ± 7.50 (7.27±14.26) 5 months 10.46 ± 7.79 (6.24±14.67)	Percentage reduction - 22.61 - 6.73	14.5/18
Kumar 2017 [3]	Intervention study	India Samastipur district 400 HHs were included For IRS Mirzapur Control in Bisanpur	Aspiration Technique and CDC light traps Baseline Monthly measurements for 15 months 5% DDT suspension	*Phlebotomus argentipes*				Percent reduction of sandfly density Month 1 81.75 (70.6–92.8) Month 2 82.40 (72.7–92.0) Month 3 9.02 (-51.9–69.9) Month 4–65.33 (-279.6–148.9) Month 5 14.97 (-20.0–50.0) Month 6 82.65 (68.4–96.8) Month 7 82.54 (70.2–94.8) Month 8 83.88 (69.2–98.4) Month 9 66.12 (49.2–82.9) Month 10 62.66 (41.7–83.5) Month 11 65.75 (39.8–91.6) Month 12 73.55 (62.1–84.9) Month 13 58.02 (12.2–103.8) The observed percent-reduction for IRS is between 4.29% - 86.77%	13/18
Coleman 2015 [30]	Intervention study	India, Bihar DDT 8 VL endemic districts, with 96 sentinel HH in 16 villages	Sand fly density/CDC light traps No baseline collection Follow–up after 1 month and 2 month post-IRS DDT	*Phlebotomus argentipes*		Total sandflies captured Baseline 0 1 month 99 3 months 122	Total sandflies captured Baseline 0 1 month 217 3 months 123	Significant differences between the IRS and non-IRS villages exist at 1 month post-IRS only (p = 0.001)	12.5/18
Chowdhury 2011/2 [21]	Intervention study	India Vaishali with 116.056 HH Nepal Sarlahi with 111.076 HH Sunsari with 5153 HH The study villages were sprayed, by the national VL-control programmes, in April–June 2008 In each area Intervention 40 HH Control 10 HH	Sand fly density/CDC light traps Baseline 2 weeks prior to IRS, At 2 weeks, 4 weeks and 6 months post-IRS India: DDT based wettable powder Nepal: Lambda-cyhalothrin	*Phlebotomus* *argentipes*		*P*. *argentipes* captured per trap India Baseline 41 (35.0–47.8) 2 weeks 3.3 (1.7–5.6) 4 weeks 7.5 (5.1–10.7) 6 months 2.3 (1.0–4.3) Nepal Sunsari Baseline 7.3 (4.9–10.4) 2 weeks 0.5 (0.0–2.8) 4 weeks 5.0 (3.1–7.7) 6 months 2.8 (1.4–4.9) Nepal Sarlahi Baseline 54.8 (47.7–62.5) 2 weeks 0.0 (-) 4 weeks 0.0 (-) 6 months 5 (3.1–7.7)	*P*. *argentipes* captured per trap India Baseline 12 (8.8–15.9) 2 weeks 13.5 (10.1–17.6) 4 weeks 14.3 (10.8–18.5) 6 months 1.25 (0.4–2.9) Nepal Sunsari Baseline 7.5 (5.1–10.7) 2 weeks 3.0 (1.1–6.5) 4 weeks 4.0 (2.3–6.5) 6 months 2.5 (1.2–4.6) Nepal Sarlahi Baseline 20.3 (16.1–25.2) 2 weeks 32.5 (27.2–38.6) 4 weeks 6.8 (4.4–9.8) 6 months 5 (3.1–7.7)	-122.26 - 75.36 - 62.96 - 31.03 17.24 6.9 - 95.73 - 87.32 - 68.29	10.5/18
Kumar 2009 [29]	Intervention study Before and after, no control	India Vaishali, Muzaffarpur, East Champaran and Saran districts 119 endemic villages in	Standard aspirator method Baseline prior to IRS 1 month and 3 months post-IRS DDT	*Phlebotomus argentipes*			In most of the districts, the incidence of kala-azar cases reduced after the DDT spray coupled with IEC activities and government efforts. There are few endemic districts like Saharsa, Saran, Muzaffarpur and Vaishali districts that have shown increase in the incidence of cases in the month of December 2007 in comparison with December 2006. Community acceptance: out of the 500 households examined, 4.8% has totally refused the spraying due to different reasons and 9.6% refused partially. After spraying the house index of *P*. *argentipes* decreased considerably when compared to pre-spray data in the study districts. Significant difference in the density of sandflies during pre- and post-spray periods in Vaishali, Saran and East Champaran districts and there was no significant difference in Muzaffarpur district. DDT is effective in controlling sand fly populations. It is suggested to strengthen the IEC activities to sensitise the community and thorough monitoring of spraying operation is essential to achieve the desired result in stipulated time. With proper monitoring and training, two rounds of DDT spray with good house coverage in all the endemic districts up to three years and proper treatment of cases are essential for total elimination of kala-azar in Bihar state.		11/18
Mukhopadhyay 1996 [22]	Intervention study	India West Bengal Intervention Hamlet 1 of Dariasudi and Chatrapara Control areas: Hamlet 2 of Dariasudi and Mulchatki	Standard mouth suction aspiration and torch light. No baseline Follow up after week 2 and week 3 post IRS, monthly measurements DDT	*Phlebotomus argentipes*, *P*. *papatasi*		Man hour densities of *P*. *argentipes* In two intervention villages Baseline 40 60 Month 1 0 0 Month 2 0 0 Month 3 0 4 Month 4 0 0 Month 5 0 0 Month 6 0 0 Month 7 0 0 Month 8 0 0 Month 9 0 1 Month 10 1 1 Month 11 0 1 Month 12 1 1	Man hour densities of *P*. *argentipes* In two control villages Baseline 26 16 Month 1 0 0 Month 2 0 10 Month 3 0 2 Month 4 0 24 Month 5 0 20 Month 6 4 16 Month 7 4 16 Month 8 7 13 Month 9 2 12 Month 10 0 10 Month 11 0 10 Month 12 1 6	The density of *P*. *argentipes* was reduced to almost zero level in all biotopes up to Sep 1994 in interventions areas. No drastic reduction in vector densities was noted in the control areas.	5.5/18
Kaul 1994 [23]	Intervention study	India Intervention Shujabad 77 HH Control Bhoipura 55 HH	Suction tubes from dark corners, ceilings, and crevices No baseline At one month’s post IRS DDT	*Phlebotomus argentipes*, *P*. *papatasi*, *Sergentomyia spp*.		Total sandfly collection Intervention: collection of 14 sand flies (0% *P*. *argentipes*, 0% *P*. *papatasi and 100% Sergentomyia spp*.).	Total sandfly collection Control: collection of 365 sand flies (91.7% *P*. *argentipes*).		5.5/18
**South America**									
Barata 2011 [14]	Intervention study Before and after, no control	Brazil, Montes Claros, Minais Gerais 10 HH	Sand fly density/CDC light traps Baseline prior to IRS Monthly for 12 months post IRS (and repeat IRS) Cypermethrin pyrothroid	*Lutzomyia spp*.			85.8% of sand flies were captures outside the houses. The sand fly fauna comprised 10 different species. *L*. *longipalpis*, *L*. *sallesi* and *L*. *intermedia* constitute approximately 90% of the fauna captured. These species were sensitive to treatment with the insecticide. The two months prior to each spraying campaign (Sep-Oct 2005 and Mar-Apr 2006) were compared with the subsequent marking periods. The results showed that, two months after spraying, significant reduction occurred only outdoors. In the second spraying period, the differences between pre- and post-spraying were significant at two months and four months after spraying. Thus, the insecticide was less effective in the first cycle (two months) and more effective in the second cycle (four months). Clear predominance of *L*. *longipalpis* (79%) both inside and outside domiciles. The authors suggest that this species has been frequently found in the home environment, and is perhaps better adapted to the presence of man and domestic animals in endemic areas of visceral leishmaniasis. The number of captured insects dropped abruptly immediately after application of two cycles of insecticide, i.e., November 2005 and May 2006, suggesting temporary efficacy in reducing sand fly population density. The authors recommend three or four insecticide sprayings every year to achieve effective control of the sand fly population.		9.5/18
**Survey**									
Hasker et al., 2012, India [25]	Survey in existing cohort study	Study site: 50 villages (200 hamlets) in the Muzaffarpur District of Bihar, India Follow-up: three annual surveys in Sep and Oct of 2008, 2009 and 2010.	In each survey, they visited all HHs and collected demographic information. Additionally, they asked whether the house had been covered by indoor residual insecticide spraying in the year preceding the survey. In each survey, they also collected information about VL in the household since the previous survey. For the first survey, they used a recall period of 1.5 years. At the time of the second survey in 2009, we also collected information about assets owned by each household, including domestic animals, and we recorded characteristics of the structure of the house and the surrounding vegetation.	*Phlebotomus argentipes*	Study population of 81.210 persons, divided over 13.416 HHs. During the study period, we registered 207 VL cases, equivalent to an average annual incidence of 72.8/100,000 population. VL was strongly associated with age; the odds of having VL was lowest for children <5 years of age and highest for children 5–14 years of age (odds ratio [OR] 2.5, 95% CI 1.5–4.0). Higher socioeconomic status was associated with reduced risk. IRS coverage was poor. In 2009 (the last year for which data were collected for the full year), only 12% of all households had reportedly been sprayed at least once.	Ownership of goats and presence of bamboo trees near the house are risk factors, but are not strong enough to warrant specific interventions. Poor housing is a stronger risk factor; thus, housing plans launched by the Indian government may positively affect control of VL.	The Musahars are known to be among the poorest of the poor, but even after we controlled for confounding by socioeconomic status, the association remained statistically significant. Some residual confounding cannot be ruled out, but other factors probably play a role. One such fac-tor could be long delays in seeking health care by Musahars, which was documented in another recent study		11/18
**Modelling studies**									
Gupta et al., 2020, India [24]	Modelling study	Study site: 33 out of 38 districts of Bihar, India Follow-up: Jan 2012 to Dec 2017	The authors compared the rate of incidence decrease in Vaishali to other districts in Bihar via an interrupted time series analysis with a spatiotemporal model, and estimated the number of cases averted by the pilot.		Changes in Vaishali’s rank among Bihar’s endemic districts in terms of monthly case numbers showed a change pre-pilot (3rd highest out of 33 reporting districts) versus during the pilot (9th). The rate of decline in Vaishali’s cases was 26th highest pre-pilot and 19th during the pilot. Model simulations suggest a median 1,071 cases were averted in Vaishali between March 2015-December 2017.		Existing interventions when applied in combination and with special attention to quality could significantly reduce incidence. Strengthening control strategies may have precipitated a faster decline in VL case numbers in Vaishali and suggests this approach should be piloted in other highly endemic districts.		13/18
Hasker et al., 2018, India [26]	Modelling study	Study site: the Muzaffarpur Health and Demographic Surveillance Site, a rural area of Muzaffarpur district, Bihar, India Data of 14.376 HHs with 91.908 persons Follow-up: from 2007 to 2015	Establishment of optimal target areas for IRS an (re)active case finding. They plotted incident VL cases on a map within a 6-months period (Jan to June or July to Dec). Buffers of 0, 50, 75, 100, 200, 300, 400 and 500 m around these cases were drewed. Recording of total population and VL case numbers diagnosed over the period in each of these buffers and beyond. Incidence rate ratios were calculated using the population at more than 500m from any case as reference category.	*Phlebotomus* *Argentipes*, *Leishmania donovani*	The risk of being diagnosed with VL within the next 6-month period was on average 45.2, 15.4, 14.6, 13.4, 9.2, 7.1, 5.9, and 5.1 times higher for those living in the same household or within 50, 75, 100, 200, 300, 400, or 500 m, respectively. There was a very strong degree of spatial clustering of VL with incidence rate ratios ranging from 45.2 for those living in the same households to 14.6 for those living within 75 m of a case diagnosed, during the previous period. Up to 500 m the incidence rate ratio was still five times higher than that of the reference category.		The findings indicate that it is important to screen also HHs within a perimeter of 50-75m from an index case. Further clustering occurs at immediate neighborhood and HH level. Covering a perimeter of 500m with IRS, seems to be a rational choice. Therefore, control interventions should also target the close surroundings of reported VL cases. Even effective IRS within a specific buffer zone would not be sufficient to prevent all cases in the next 6-month period for several reasons. First of all IRS would not prevent VL in a person already infected but still in the incubation phase, secondly because people may also be infected in outdoor locations.		13/18
Stauch et al., 2014, India [27]	Modelling study	1970 to 1986	Usage of a previously published VL model that has been used to investigate emerging resistance against antimonial treatment. Development of a system of ordinary differential equations to model the transmission dynamics of *L*. *donovani* between sand flies and humans on the Indian subcontinent. Investigation of transmission thresholds dependent on measures reducing the sand fly density either by killing sand flies (e.g., indoor residual spraying and long-lasting insecticidal nets) or by destroying breeding sites (e.g., environmental management).	*Leishmania donovani*	The elimination of VL is possible if the sand fly density can be reduced by 67% through killing sand flies, or if the number of breeding sites can be reduced by more than 79% through environmental management.	Treated nets and to a minor extent IRS, predominantly kill sand flies that are about to transmit the infection, whereas breeding site control generally reduces the number of flies, regardless of whether they reach an age where transmission occurs.	Reduction of the vector’s life expectancy is more effective than a reduction of the vector’s breeding site capacity. LLIN are a highly effective intervention tool because treated nets can be considered as baited traps that kill predominantly sand flies that are about to transmit the infection. Three major reasons may limit the effectiveness of LLIN: (1) inappropriate usage of LLIN by man, (2) changed and/or alternative feeding or resting behaviour of the vectors and (3) vector adaptation or habituation against insecticidal substances. Destroying breeding sites of *P*. *argentipes* is a promising tool for intervention and should also prevent re-emergence of infection after local extinction. Resistance against DDT continues to spread and cross-resistance may emerge (e.g., in anophelines, the so-called knockdown resistance, a DDT/pyrethroid cross-resistance, is commonly found. Thus, IRS may only be a transient measure to effectively reduce sand fly density. Integrated vector management, which combines different vector control measures, could be an effective approach to overcome the limitations of independently applied vector control strategies.		13/18

Similarly, when analysing the time frame of the studies, the studies of the Regional Initiative cluster between 2009 and 2020, with two studies from the 1990s in the region [22,23], but before the Regional Initiative. The study from Brazil was conducted in 2011 (Barata).

Also, the modelling studies and the included survey, are geographically in the Regional Initiative [24–27], and conducted in the 2010s.

*Clustering of authors and groups of authors*. Most studies, including the modelling studies [24,26,27] (India) and the included survey [25] were either directly conducted with a group of authors affiliated to WHO/TDR, or have been involved with this group and the Regional Initiative (Tables 1 and S1). For the geographical area of the Regional Initiative only the two studies from the 1990s [22,23] were not conducted by the group of authors and/or affiliated to this group. Funding has been provided often by WHO/TDR directly, but also by other funding agencies, such as the Bill and Melinda Gates Foundation, European Union funded programmes, and the Indian Council for Medical Research.

*Methods used by the included studies*. Of the 17 included studies assessing effectiveness of IRS, the five cRCTs [4,15,16,17,18] and the RCT [19] clearly specify the methodology used, and in detail. However, no internationally recognised standard for the methodology is specified—such as the “extension to cluster randomised trials of the Consort 2010 statement” [28]. Similarly, the 11 intervention studies are not specifying any standard used, and vary in study design, with Intervention-Control designs, Intervention-Intervention designs, and Before-and-After designs.

Furthermore, overall methods in the studies vary as well, since multiple study arms are applied, mixing different methods. As an example, Chowdhury [21] mixes six methodological tools, with formal interviews with district VL officers responsible for the VL control programme, structured observations of the spraying teams-assay-based monitoring of the bio- availability of insecticides, quantification of insecticide concentrations on sprayed walls, tube bioassays for susceptibility of local *Phlebotomus argentipes–*and monitoring of sandfly densities. Some studies, Huda [4] for example, mix different vector control interventions in one study arm, with the use of IRS and Temephos.

The survey included [25] and the modelling studies [24–27] specify their respective methods, however no standards for methods are used. Also, the focus of each of these studies is different. Hasker [25] is embedded in an ongoing VL cohort study and establishes risk factors for developing VL as a disease, including IRS. Gupta [24] estimates incidence decreases with spatiotemporal models, in relation to an “intensified control strategy”, including the use of IRS. Hasker [26] aims to establish the most appropriate “buffer zone” for perifocal application of IRS, and Stauch [27] estimates transmission thresholds in relation to application of interventions, including IRS.

*Sample sizes and follow up periods of the included studies*. In general, sample sizes vary considerably between the studies:

Since most cRCTs and RCT have multiple study arms, samples sizes vary also between the study arms (Tables 1 and S1). Huda [4] for example operates in a total of 8143 households (HHs) with 36,869 people. However, the study follows up on VL cases, identifying different levels of VL transmission, and intervening with study arms of “House-to-house active search for cases with VL and PKDL, implementation of IRS with deltamethrin and deployment of larvicide Temephos”, or “fever camps plus installation of durable wall lining impregnated with deltamethrin” or “fever camps plus impregnation of existing bednets with a slow-release insecticide tablet”. Also Cone bioassay tests are performed. The HH sandfly density measurement however is performed in 36 HHs only, in each study arm.

The number of surveyed HHs per cluster is highest with 24 clusters with 120 HHs each [17]. The RCT has 264 intervention and 92 control HHs [19].

The intervention studies also have massive sample sizes for the overall study and a smaller subset of HHs surveyed for entomological assessment, for example Chowdhury [21] operates in districts in India and Nepal, with 116056, 111076 and 5153 HHs respectively, however the entomological assessment was implemented in 40 HHs in each intervention and 10 HHs in the control. Different are the early intervention studies from 1994 [23] and 1996 [22], with smaller intervention and control sites, with for example 77HHs for the intervention and 55 HHs for the control [22].

Follow up periods and measurement vary also considerably, with earlier intervention studies typically measuring a baseline and a follow up measurement one month post IRS [23].

The cRCTs and the RCT have multiple measurements, mostly a baseline and four to five follow up measurements post IRS, up to 12 months (e.g. Huda [4]). However, Chowdhury [15] measures up to 22 months post IRS with nine follow up measurements.

The modelling studies and the survey follow different methodologies and no entomological measurements have been implemented [24–27].

*Outcome measures and sampling methods of the included studies*. Outcome measures varied according to the objectives of the included studies, and study arms (Tables 1 and S1). For entomological indicators the most commonly used indicator is the total number of *Phlebotomus argentipes* caught, sometimes total number of sandflies caught. Very few studies further distinguish into female and male *Phlebotomus argentipes*, and even fewer analyse counts of gravid *Phlebotomus argentipes* females or fed *Phlebotomus argentipes* females. Percentage reductions between intervention and control sites are mostly calculated.

Sandfly captures are consistently performed with CDC light traps, older studies, such as Das [18] also use manual mouth aspirator methods and compare the methods. Even older studies, such as Kaul [23] and Mukhopadhyay [22] are relying only on manual aspirator methods.

As for human disease indicators, these are only very seldom measured, and the indicators vary: Huda [4] performs house to house searches for fever cases of more than 2 weeks, and/or for Post Kala Azar Dermal Leishmaniasis (PKDL) skin lesions. Kumar [3] also measured human disease with active case detection/ house to house searches and follow up of fever cases with 39-aminoacid–recombinant kinesin antigen (rK39) rapid diagnostic tests. For the survey [25], identification of VL cases was key objective of the wider cohort study, in which the survey was embedded. VL cases in the cohort study were defined as “*a combination of a clinical history typical for VL (fever of >2 weeks’ duration*, *lack of response to antimalarial drug treatment)*, *a positive result by the rK39 rapid diagnostic test*, *and a good response to specific VL treatment*, *with or without confirmation of parasites*. *Each case reported was verified from medical records by a study physician*”. The modelling studies used available surveillance data for human disease indicators.

*Insecticides used by the included studies(28)*. Also, the insecticides used in the studies vary, mostly in line with insecticide development over time and specific to countries (Tables 1 and S1).

DDT is used in the earlier studies in India [22,23], DDT continues to be used in Indian studies only (Joshi [17] for the India arm of the study, Kumar [29], Chowdhury [21] for the Indian arm of the study, Coleman [30], Kumar [31], Poché [32]). Mandal [33], and Kumar [3] switch during the studies from DDT to alpha-Cypermethrin. It needs to be noted that DDT has been banned in Bangladesh since 1998 as it is an environmental hazard [20].

Alpha-Cypermethrin is also used in Bangladesh [15], Brazil [14] and Nepal (Joshi [17] for the Nepal arm of the study, Das [18]).

Deltamethrin is used in Bangladesh only (Joshi [17] for the Bangladesh arm of the study, Chowdhury [16], Chowdhury [20], Banjara [19], Huda [4]). This has been tested in more recent studies, as there was a delay in getting approval for using deltamethrin for sandfly control and reintroducing it through the national programme [20].

Lambda-cyhalothrin is only used in Nepal (Chowdhury [21] for the Nepal arm of the study)

*Quality assessment of the included studies*. (Table 1: Summary IRS results and S1 Table: Evidence Table)

Quality analysis showed, when measuring against the CONSORT criteria that the RCTs only scored medium high scores, between 11 and 18 of 25 CONSORT criteria are met. Mostly trial registration criteria are not met. But also, processes of randomisation are not fully explained. For the intervention studies, most studies scored between 10.5 to 15.5 of 18 modified CONSORT criteria. The early studies Kaul [23] and Mukhopadhyay [22] only met 5.5 of the 18 criteria.

### Analysis of results

#### Entomological indicators

*cRCTs and RCT*. In the group of cRCTs and RCT, the studies show mostly very positive results in relation to the main indicator used “total *Phlebotomus argentipes”* caught, and also considering the different insecticides used for IRS (Table 1: Summary IRS results).

For alpha-Cypermethrin, in the cRCTs and RCT applied in Nepal only, Chowdhury [15] showed reductions of the vector between 14 and 80%, with a considerable effect even at the end of the measurement period (two rounds of IRS). These data were established over a period of up to 22 months, with measurements for the indicator available for up to 15 months. Das [18], with measurements up to five months post IRS, showed *Phlebotomus argentipes* density decreasing from 11.0 to 0.6 per house per night, labelling therefore IRS highly effective for reducing vector density. Joshi [17] for the Nepal arm of the study shows a reduction of total sandflies captured of 52 and 53% in two different sites.

Deltamethrin is used in Bangladesh only, Joshi [17], for the Bangladesh arm of the study, shows a reduction of 94% of total sandflies caught. Chowdhury [16] showed Rate Ratio reductions to 0.15 after 2 months post IRS compared to control, 0.24 after 5 months. The positive effect of IRS was not detectable any more after 12 months. Huda [4] applied IRS with simultaneous treatment of breeding sites with Temephos, this combination showed even a 74% reduction of female *Phlebotomus argentipes* captured after 12 months. However, Banjara [19] showed only a reduction of 25.13% of total sandflies caught, after one month. The effect of IRS was not detectable any longer in subsequent measurements.

DDT has only been used by Joshi [17] in India, with a percentage reduction that varied from 52 to 124% of total sandfly captures between sites, with measurements five months post IRS.

*Intervention studies*. As for DDT (Table 1: Summary IRS results), this is first used in two earlier studies in India [22,23]. These two smaller intervention studies, with control villages, resulted in a reduction of more than 90% of total sandflies caught in the intervention villages in both studies, with measurable effects up to one year post IRS [22,29], in a before and after study, shows a positive effect one month post IRS, in three of four of the study districts: *“significant difference in the density of sandflies during pre- and post-spray period in Vaishali*, *Saran and East Champaran districts (p <0*.*05) and there was no significant difference in Muzaffarpur district (p> 0*.*05)”*. Chowdhury [21] for the Indian arm of the study, shows reductions of up to 75.36% one month post IRS, which were not sustained afterwards. Coleman [30] also shows a difference between intervention and control villages and total sandflies captures (total sandflies captured at one month post IRS 99 for the intervention villages, 217 for the control villages), and the effect was not observed after one month. Kumar [31] shows over a period of 13 months post IRS, percent reductions of sandfly density between 4.29% - 86.77%. Poché [32] reports a short-lived positive effect of IRS, results “*suggest relative Phlebotomus argentipes abundance within IRS treated and untreated villages do not differ significantly*”. Mandal [33], and Kumar [3] switch during the studies from DDT to alpha-Cypermethrin: reporting for the former for the initial IRS percentage reductions of total *Phlebotomus argentipes* captured of 34.1% after two weeks, 25.9% after one month and only 14.1% after three months [33] and similar results by Kumar [3].

Alpha-Cypermethrin was used in these last two studies [3,33] for the subsequent rounds of IRS, with percentage reductions of 90.5% after two weeks, 66.7% after one month and 55.6% after three months [33]. Kumar [3] summarised the follow up of up to three months post IRS as “*a significant difference was observed in Phlebotomus argentipes densities collected in sprayed and unsprayed villages during the 15 days*, *1 month*, *and 3 months post-IRS collections (t = 4*.*6; P <0*.*01)*.*”*

Alpha-Cypermethrin is also used in Brazil [14], targeting predominantly *Lutzomyia longipalpis*, with positive effects on the vector indoors–and outdoors.

Deltamethrin is used in the intervention studies in Bangladesh only [20], with some reductions of total *Phlebotomus argentipes* caught, of 22.61% after one month, and 6.73% after five months.

Lambda-cyhalothrin is only used in Nepal (Chowdhury [21] for the Nepal arm of the study), with good percentage reductions in one study area, between 95.73% two weeks post IRS, reduced to 68.29% after 6 months. However, these results were not repeated in another study area, where a sustained percentage reduction was not observed.

*Surveys and modelling studies*. The studies in this group are very heterogenous.

The survey included [25] (Table 1: Summary IRS results), embedded in a VL cohort study, establishes that reported IRS coverage is poor, and may be a contributing factor to VL incidence (only 12% of all households had been reportedly sprayed).

For the modelling studies, Gupta [24] estimates incidence decreases in relation to an “intensified control strategy”. Part of this strategy is IRS, in this case first using DDT, followed by alpha-Cypermethrin. The authors show that *“model simulations suggest a median 1*,*071 cases (IQR 849–1*,*333) were averted in Vaishali between March 2015-December 2017*.*”* Hasker [26], analysing the most appropriate “buffer zone” for perifocal application of IRS, concludes that *“covering a perimeter of 500m with IRS*, *seems to be a rational choice*”.

Stauch [27], estimating transmission thresholds in relation to application of interventions, including IRS, however, considers “*thus*, *IRS may only be a transient measure to effectively reduce sand fly density*”.

*Human disease indicators*. The studies only seldom measure human disease indicators (Table 1: Summary IRS results), or present surveillance data for human disease indicators. These data are not in direct comparison to IRS intervention, or case numbers are too low for clear measurements.

Huda [4] compares case finding with different methods, without comparing the results directly to IRS intervention only. The authors recommend for programmes to introduce elements of active case finding through index-based fever camps.

Kumar [3] also measured human disease with active case detection/house to house searches and concluded that the elimination strategy achieved its goals “*the case reduction rate was increased from 22*.*6% in 2014 to 58*.*8% in 2016*.*”*

Hasker [25] studies a cohort in India and follows up on VL cases, the study determines annual incidence of the population, without prospectively analysing IRS as an intervention. However, IRS is established as a potential protective factor of VL.

*Crosscutting results*. The studies show a trend over time of insecticide use (Table 1: Summary IRS results). DDT is used in India only, over time, very broadly speaking, the studies show a reduced effect on vector populations, from Kaul [23], Mukhopadhyay [22], followed by Kumar [29], Chowdhury [21], Coleman [30], Kumar [31] and Poché [32]. Finally, in the studies by Mandal [33] and Kumar [3], during the studies, the insecticides are changed in the overall programmes, where the studies are embedded in, from DDT to alpha-Cypermethrin.

Alpha-Cypermethrin and Deltamethrin are now the insecticides mostly applied in studies (for the former Joshi [17], Das [18] and Chowdhury [15] (all Nepal), for the latter Joshi [17], Chowdhury [16] and [20], Banjara [19] and Huda [4] (all Bangladesh).

Many studies have several study arms, particularly also assessing insecticide bioavailability and residual activity, using bioassays. These study arms follow the recommendations of the WHO/TDR monitoring and evaluation tool kit for IRS [34]. These study arms, but also study arms to train personnel for quality delivery of IRS—including qualitative study arms, show the importance of quality assurance for IRS. Most of the studies of the Regional Initiative include these different methods, for example for bioassays Kumar [3], Mandal [33], Huda [4], Banajar [18], Chowdhury [20], Kumar [31], Coleman [30], Chowdhury [16,21] and Joshi [17].

Combination of different interventions are also evolving over time in the included studies. Whereas early studies, Kaul [23] and Mudhopadhyay [22], typically test only one intervention, in this case the use of DDT. This is also the case for later studies, as for example Chowdhury [15] and Mandal [33], with study arms examining different interventions, but mostly not adding and combining different interventions in one study arm.

However, later studies also often try combinations of interventions, for example Huda [4] adds Temephos to the use of IRS in one study arm, but also tests different ways to identify VL cases, with one study arm combining fever camps and installation of durable wall lining.

*Study design evolves over time*. Clearly, early intervention designs, as applied by Kaul [23] and Mukhopadhyay [22] are replaced by cRCTs, with the first cRCT for vector control and VL by Joshi [17]. However, intervention designs continue with later studies, more embedded in existing programmes, and assessing programmes on multiple levels [3,20,21,30].

*Gap analysis*. When analysing gaps, following hierarchy of evidence principles, and trend over times, a gap analysis of studies assessing effectiveness of IRS for the control of VL shows the following elements:

- From 2009 onwards an increase in numbers of studies is available
- There is a trend over time from intervention studies to more complex studies, with five cRCTs and one RCT included.
- When looking at study questions, these are highly variable, and not always focused on the effectiveness of IRS only.
- When looking at study arms, there are increasingly studies mixing different combinations of interventions. On the other side, there are fewer studies, assessing IRS only. This is more important in the different transmission scenarios, particularly in the Regional Initiative, now mostly entering elimination of VL.
- Human disease parameters are seldom measured.
- Studies are more often embedded in ongoing programmes, with the data available in the programmes.
- When looking at geographical coverage, most included studies were conducted in the countries of the Regional Initiative, namely Bangladesh, India and Nepal (16 of 17 effectiveness studies, and of all studies 18 of 21). There is only one study from Brazil [14]. Notably absent are studies from other VL affected countries, like South Sudan and Sudan. Also absent are studies from the other South-East Asian countries reporting VL cases and sandfly vector population.

## Discussion

### Discussion of key results

Before discussing the key results it needs to be highlighted that this systematic review has several limitations: As there is relatively few primary research in this field available the studies are very heterogeneous and the comparability is limited. The effectiveness of the IRS intervention study arm is often not clear to disentangle from other study associated factors, the vectors, but also other biological factor are heterogeneous in different settings and populations. All together hindering the establishment of a consistent picture. However, effects of IRS on vector populations are positive, confirmed in terms of effectiveness and by the availability of studies: in general, overall results confirm, that IRS has good immediate effects on vector populations of *Phlebotomus argentipes*. The results from five cRCTs and the RCT included in the systematic review, clearly underline this, using Deltamethrin and alpha-Cypermethrin, for Bangladesh, India and Nepal. These results are also confirmed by two intervention studies, using alpha-Cypermethrin, in ongoing programmes [3,33].

The role of IRS for VL control has been discussed consistently (Picado 2012), and with the results of the included studies, and in very general terms, there is a clear and measurable effect of IRS on the key vector *Phlebotomus argentipes* for the three countries of the Regional Initiative documented through the included studies.

Effects of IRS on VL incidence is not well established. Studies have not included human transmission indicators; this would also be technically difficult. However, some data for human transmission are coming from studies embedded in programmes, although these cannot be directly related to IRS interventions only. Using VL as outcome in future studies will become even more difficult as the disease incidence has become very low in the South-East Asian region.

As a further element emerging in the included studies, and mentioned by most studies, quality of delivery is key for IRS. One of the elements for quality of delivery is insecticide bioavailability and resistance. There seems to be an acceptance to include bioassays in studies, most of the newer studies include this element. This conforms with a guidance issued by WHO/TDR [34].

Also, several studies include “inspection” and “training” for delivery of IRS, by programme staff. Kumar [3] concludes that “*the success of a control program is directly related to the quality of the staff*. *Therefore*, *human resource development is necessary for all control programs*.”

As a further element of quality delivery, timing of intervention may play a role. There seems to be agreement to apply IRS in the Regional Initiative pre-Monsoon, as also outlined by WHO/TDR [34].

As a further element of studies, the measurement technique is also evolving over time. Whereas early studies used standard aspiration techniques, CDC light traps are now the established way of measuring sandfly populations.

Furthermore, the topic of combinations of different vector control methods emerged from some studies. Again, since the combinations including IRS were only seldom used in the studies, and no consistent assessment of different combinations has been done, this systematic review cannot give an answer about improved effectiveness, when IRS is combined with other methods. But to mention one study, Huda [4] adds to IRS with Deltamethrin the larvicide Temephos, targeting breeding sites. The combination is thought to be promising, also with the mentioned prolonged effect. However, Huda shows even better results including durable wall lining (DWL). Clearly, combinations of best possible vector control interventions may be useful, and even synergistic, and need appropriate studies. The choice of combinations of interventions may even be more dependent on local situations, as entomological characteristics, human behavioural factors, than actual synergies between different methods.

This goes in line with a recent meta-review, with several vector control interventions showing some effect on VL vectors [8]: the interventions included IRS, the use of ITNs, insecticide-treated curtains (ITCs) (including insecticide-treated house screening), insecticide-treated bedsheets (ITSs) and insecticide-treated fabrics (ITFs) (including insecticide treated clothing) and DWL and other measures to protect houses. Environmental modifications (EVM) were evaluated, also control of the reservoir host, and strengthening vector control operations through health education. The multitude of possible interventions and combinations of interventions show a further need of studies.

### Discussion of insecticides used in the studies

For Deltamethrin, the positive effect, with very high suppression of vector populations with up to and over 90% one month post IRS was shown in three cRCTs in Bangladesh [4,16,17]. There is also a prolonged effect, strengthened by simultaneous treatment of breeding sites with Temephos [4]. This effect can be shown medium long term, with good results up to 12 months post intervention.

However, in a further RCT [19] and one intervention study [20], in Bangladesh, deltamethrin did not show a strong effect, and on its own, not a prolonged effect.

For alpha-Cypermethrin, very positive results are confirmed by two cRCTs [15,18,24], with intermediate results by a further cRCT [17]. Additionally, two intervention studies confirm the result with a good effect up to three months [3,33].

In Brazil, with a different predominant vector (*Lutzomyia longipalpis*) alpha-Cypermethrin was successful indoors and outdoors, however, the study was only comparing “before and after” measurements in the same houses.

DDT has demonstrated limited effects, waning over time, as confirmed by nine intervention studies, all conducted in India. This can be tracked to 2009 [29], and is essentially different in studies reported in the 1990s [22,23]. Two intervention studies “switch” insecticides from DDT to alpha-Cypermethrin [3,33]. However, only one cRCT used DDT, for the study arm in India [16], with very good results.

The use of DDT in the studies has been limited to India, but the limited effect has been already established by Coleman [30]: "*in India*, *widespread resistance to DDT*, *the insecticide used*, *combined with poor quality assurance of IRS and limited entomological surveillance*, *is hindering the VL elimination effort*.*"*

Lambda-cyhalothrin has been used in one intervention study only, with positive and negative results in different study areas in Nepal (Chowdhury [21] for the Nepal arm of the study).

Cleary, the choice of insecticides for IRS is key to success, related to resistance development and regulations in countries. Guidance exists through WHO/TDR [34].

### Discussion of level of evidence

Policy recommendations should be evidence based [35]. Hierarchy of evidence with its categorisation plays a role. On a positive note, five cRCTs and one RCT, and 11 intervention studies underpin the research question.

However, and limiting the key results of this systematic review, when considering further questions of different insecticides used, the lack of studies for best combinations of interventions, and the lack of geographical coverage, a need of further studies clearly arises. This is even more important considering the different vectors in countries affected by VL.

Furthermore, other vector control interventions, as outlined by Montenegro [8], should be systematically reviewed. This would help to estimate the level of effectiveness of each vector control intervention and so inform to 1) Determine a priori best possible combinations of interventions for further studies, and 2) Determine policy recommendations with the current available knowledge.

Studies should take different transmission levels into account, considering that for the Regional Initiative recommendations may be different, with VL being eliminated as a public health problem, compared to a situation with more VL transmission.

There is also a concern, since none of the donor of the regional initiatives were come forwarded to invest in generating the evidence on every sector of visceral leishmaniasis except few diagnostic and drug manufacturers. Due to this fact most of the studies included in this systematic review are related to one group of authors, mostly related or funded through WHO/TDR. It would be highly recommendable to stimulate more independent generations of researchers to be involved in further studies.

## Conclusions and recommendations

This systematic review has answered the objective to systematically review all available evidence for the control of the key vectors of VL through IRS:

- Effects of IRS on vector populations are positive, confirmed in terms of effectiveness and by the availability of studies. IRS continues to be an effective intervention to control *Phlebotomus argentipes*. Cost-effective ways of using IRS in combination with another vector control technique like ITNs, along with case detection in the community needs to be designed for the post-elimination era in South-East Asia when the disease burden is very low.

- Deltamethrin and alpha-Cypermethrin reduce total sandfly counts, and/or *Phlebotomus argentipes* counts by up to 95% after one month. Prolonged effects are not regularly seen. DDT has been used in India only: whereas in the 1990s a good effect could be measured, this effect waned over time. Two intervention studies, published in 2019 and 2020, embedded in larger programmes, replaced DDT with alpha-Cypermethrin throughout the study.

- Constant monitoring of insecticide resistancies and quality delivery of IRS emerge as key issues for programmes. No human transmission data are available to directly relate an effect of IRS–although modelling studies confirm the effect of IRS on human transmission.

- The varied results obtained in the different studies, makes it difficult to provide a clear recommendation on when and how long (in the shorter and longer terms) IRS should be implemented, especially in regions where the disease burden is very low. Following recommendations from WHO/TDR [34] and from the analysed studies; in terms of when to apply IRS during the year, it is recommended to coincide with the build-up of vector populations (March or April in the South-East Asia region) and before the onset of VL transmission (from June to October in the South-East Asia region). In terms on how long IRS should be applied in the shorter term, within a particular year, it is important to consider the application of IRS in an integrated vector management approach, which could lengthen the effect of IRS. In this sense, IRS should be applied in two rounds over a period of 12 months. Of course this could vary from country to country and necessary adjustments should be consider. In regard to the longer-term continuation of the annual IRS application, especially in the current low burden scenario in South-East Asia, we recommend taking cautious approach considering scarcity of resources available for VL control at the country level, especially when there are competing priority programs [36]. As the Regional Initiative is transitioning through the consolidation to the maintenance phase aiming at achieving long-term elimination, a vertical and blanket approach may not be feasible and sustainable. A nationwide integrated approach is needed combining case finding, treatment and vector control. This needs to be continued for a longer period of time, supported by routine disease and vector surveillance to prevent resurgence of VL in the communities.

- Combinations of different interventions are not sufficiently and systematically researched. Also, studies were not conducted in several countries with VL transmission, including different transmitting vectors.

- Systematic reviews for all available VL vector control interventions should be developed, and a process of determining best practice health policy recommendations should be initiated.

## Supporting information

S1 Table Evidence table. Summary of studies included in the systematic review.
(DOCX)
