# Supplementary material for: Indoor residual spraying for the control of visceral leishmaniasis: A systematic review
Source: PLoS Negl Trop Dis. 2022 May 19;16(5):e0010391. doi: 10.1371/journal.pntd.0010391 (PMC9159594; doi:10.1371/journal.pntd.0010391)
Supplement: S1 Table — Summary of studies included in the systematic review. (DOCX) [file pntd.0010391.s001.docx]

| **Author, year, country** | **Study type/ Methodology** | **Sample size/ Follow-up** | **Study arms /Interventions in detail** | **Species of Vector/ Species of parasite** | **Results:**  **Human outcome indicators** | **Results:**  **Entomological outcome indicators** | **Conclusions by the authors** | **Limitations described** | **Quality assessment scores by the authors** |
| --- | --- | --- | --- | --- | --- | --- | --- | --- | --- |
| **cRCTs** | | | | | | | | | |
| Huda et al., 2019, Bangladesh [1] | cRCT | Study site: VL-endemic villages with a recent VL case in highly VL-endemic upazilas (subdistricts), Fulbaria and Trishal of Mymensingh district  Total number of HHs: 8143 with 36.869 people  Follow-up: from Sep 2015 to June 2017: 4 months of pre-intervention, 2 months of intervention and 12 months of follow-up activities.  Sandfly density survey: 2–3 days before starting the intervention and follow-up surveys at 1, 3, 6, 9, and 12 months. | Selection of areas for index case–based intervention:  VL incidence at the union level was calculated to stratify the study areas for index case–based intervention activities. They identified three pairs of unions with similar VL incidence and randomly assigned each of the three interventions to each pair. A pair of unions with similar VL incidence was selected as control group.  Intervention areas: Trishal  Control areas: Fulbaria  Selection of HHs for entomological assessment:  Random selection of three index case–based intervention clusters from each arm for entomological efficacy trials.  12 households from each of the three clusters in each intervention arm were selected randomly, which yielded 36 households in each intervention arm. Three different matched control clusters with 36 HHs were selected from the control group.  Experimental interventions:  No kala-azar transmission activity: House-to-house active search for cases with VL and PKDL, implementation of IRS with deltamethrin, and deployment of larvicide (Temephos 50 EC, 5 mL/10 L) (Limbate 50 EC, D Limit Agro Product Limited, reg. no. 163, Bangladesh) at suspected sandfly breeding places in 60 households around the index household.  Installation of fever camps plus installation of DWL impregnated with insecticides (deltamethrin, 170 mg a.i./m^2^) in 60 households around the IC’s household.  Installation of fewer camps installation of fewer camps plus impregnation of existing bednets  with a slow-release insecticide tablet.  Cone bioassay test:  in 12 randomly selected households out of 36 households for sandfly density measurement  per intervention at 1, 3, 6, 9, and 12 months. | *Phlebotomus argentipes* | Findings: 49 VL index cases during the study period from 28 villages in the study area, of which 18 were NKTA in 11 villages, 12 were FC + DWL in 10 villages, and 19 were FC + ITN in seven villages.  Fifteen (29%) cases in the camp in the FC + DWL arm were referred to the upazila health complex for further management, five of whom were confirmed as PKDL. The FC + ITN arm also referred 19 (37%) of the cases to the upazila health complexes, of whom one was confirmed as VL and two as PKDL. | The estimated mean reduction of sandfly count per HH attributed to IRS plus larvicide and installation of DWL were, respectively, −1.04 (−0.63, −1.42) and −0.56 (−0.33, −0.77) at 12-month follow-up. The mean reduction of female P. argentipes density in the ITN arm ranged from −6.10 (−3.04, −9.19) to 0.81 (0.01, 1.60).  The adjusted model showed a significant reduction in the incidence rate of female P. argentipes sandfly count in the NKTA and FC + DWL arms compared with the control arms up to 12 months post-intervention except at the 9-month follow-up. The reduction in the incidence rate of female P. argentipes sandfly count was 74% and 79% at 12-month follow-up in the NKTA and FC + DWL arms, respectively. The adjusted intervention effect of ITN was statistically significant on reduction of the incidence rate of female P. argentipes sandfly count up to 3 months post-intervention but not beyond follow-ups.  Efficacy based on WHO cone bioassay: The corrected mortality of P. argentipes was 95.1%, 51.7%, and 48.5% on the DWL surface walls, insecticide-treated bed-nets, and IRS surface walls, respectively, at the 12-month follow-up. | Index case–based FCs are more useful for the early detection of VL and PKDL cases than house-to-house search in NKTA in the consolidation phase of the VL elimination program.  Durable wall lining has a stronger and longer lasting killing effect for 12 months or possibly longer than IRS and ITN.  Durable wall lining and IRS plus larvicide exert protective efficacy for 12 months and possibly more, whereas ITN (K-O Tab 1-2-3 impregnation) has inconsistent efficacy.  Combining IRS with larviciding in NKTA was found to be effective for 12 months and perhaps longer, as it lasts at least twice as long as the well-documented 5–6 months with IRS alone.  Index case–based camps were effective to detect VL and PKDL cases. Therefore, index case–based FC is a sensitive method to detect cases when they are few and would be missed by the classical no kala-azar transmission activity (NKTA).  They recommend index case–based FC for ACD combined with DWL or IRS plus larvicide for sandfly control during the consolidation and maintenance phases of the VL elimination program. | They had fewer index cases than planned, which meant that only two of the three arms (NKTA and FC + ITN) had the minimum calculated 18 index case–based interventions.  They could not have an epidemiological end point because of the currently low incidence of VL in Bangladesh; therefore, they could not measure the effects of reduced vector densities on parasite transmission and new infections and cases. | 17/25 |
| Chowdhury et al., 2017, Bangladesh [2] | cRCT | Study site: Fulbaria upazila (sub-district) of Mymensingh district  3079 houses from 11 villages divided into 10 sections with 6 clusters per section  Baseline measurement of vector density  Follow up: 2, 4, 5, 7, 11, 14, 15, 18 and 22 months after intervention | Five HHs from each cluster (300 HHs) were selected by simple random sampling for measuring sand fly densities at 3 weeks before intervention (baseline survey) and in the follow-up surveys.  Intervention arms:  1) IRS: The inside of the all house walls were sprayed with Alpha Cypermethrin 5 WP  2) LLIN were donated to each HH of selected clusters according to their sleeping arrangement.  3) Slow release insecticide treated bed nets (KOTAB): In the selected HHs of study villages, bed nets were impregnated with slow release insecticide (deltamethrin) tablet K-O TAB 1-2-3  4) Insecticide spraying in the possible breeding places (OUT)  5) And the following combinations were implemented in the selected HHs: IRS and LLIN, IRS and KOTAB, IRS and OUT, LLIN and OUT and KOTAB and OUT.  6) Control: no intervention  Sand fly collections using CDC light traps | *Phlebotomus argentipes* |  | A total 17,434 sand flies were collected during the entire study period including baseline and the 9 follow-up surveys. Of all sand flies, 53.75% were *P. argentipes.*  At baseline, the average *P. argentipes* density per HH was 10.57 in the control arm and 7.3 to 11.53 in the different intervention arms. *P. argentipes* sand fly densities in most of the intervention arms were significantly lower than the control arm at different follow-up measurements except OUT.  IRS: Two consecutive cycles of IRS were effective  in significantly reducing sand fly vector densities for more than 12 months (January 2013 to March 2014). The reduction of sand fly vector densities was 14% to 80% at the different points of measurement and the rate ratio (RR) of *P. argentipes* sand fly counts after and before the intervention was 0.86 and 0.20 up to a 15 months period.  The LLIN was effective from the second follow-up onwards throughout the study period. The adjusted model for LLIN showed that the vector density reduction was 9% to 78% and the rate ratio was between 0.91 and 0.32 for two years.  In the KOTAB arm, a significant sand fly density reduction was observed only at the third, sixth, seventh and eighth follow-up measurements.  For the other combined intervention arms: The combination of IRS with LLIN (IRS+LLIN) or with KOTAB (IRS+KOTAB) and with outdoor spray (IRS+OUT) showed statistically significant *P. argentipes* reductions throughout the study period except at the second follow-up for IRS+KOTAB.  The adjusted model showed that the effects of IRS+LLIN and IRS+KOTAB were 23.0% density reduction to 85.0% and 16.0% to 86.0% respectively. The combination of LLIN with outdoor spray (LLIN+OUT) was found to be effective throughout the study period at a highly significance level. The reduction of *P*. *argentipes* sand fly density in this case was 26.0% to 86.0%.  The combination of outdoor spraying with KOTAB (OUT+KOTAB) had no statistically significant effect on density reduction of *P*. *argentipes* sand fly counts in most of the follow-up points.  For the Sand fly mortality bioassays: The mortality for LLIN was 82.59% at 20 months of use. The mortality on K-O TAB 1-2-3 impregnated nets dropped from 88.37% at 3 months to 69.12% at 20 months after use. | The combination of different approaches leads to better results than single approaches in reducing the vector population.  IRS is however challenging in terms of operational complexity and cost and it is difficult to maintain a uniform quality of spraying. It was observed in India and Nepal that when IRS was applied by the research team under well controlled conditions, it was found to be very effective against VL vectors but when it was delivered by the national program the efficacy dropped significantly.  The combination of LLIN and OUT (outdoor spraying of vector breeding sites) was the most efficacious measure among the different tools tested. Operationally, this is much easier to apply than IRS. | The study did not evaluate the relationship between vector density (males plus females) and leishmaniasis incidence, which the research indicates that it should be investigated. | 18/25 |

**S1 Table. Evidence table.** Summary of studies included in the systematic review.

| **Author, year, country** | **Study type/ Methodology** | **Sample size/ Follow-up** | **Study arms /Interventions in detail** | **Species of Vector/ Species of parasite** | **Results:**  **Human outcome indicators** | **Results:**  **Entomological outcome indicators** | **Conclusions by the authors** | **Limitations described** | **Quality assessment scores by the authors** |
| --- | --- | --- | --- | --- | --- | --- | --- | --- | --- |
| Chowdhury et al, 2011/1, Bangladesh [3] | cRCT | Interventions were conducted in Fulbaria subdistrict, Mymensingh District.  Four villages were  randomly selected from the 20 villages, each village was divided into six geographic clusters (24 clusters). 120 HHs were selected for vector collection.  Pre-intervention vector collections: in  October 2006.  Follow up: in  December 2006, January, March, April, and October 2007. | Pre-intervention deltamethrin susceptibility testing:  They used sand flies collected from randomly selected HHs.  Susceptibility was tested in tube bioassays.  Intervention groups:  (1) IRS with deltamethrin with a target concentration of 20 mg active ingredient/m2.  (2) PermaNet 2.0 ITNs were distributed to all houses in the selected clusters to provide space for all family members to sleep under a net. A total of 489 nets were distributed to 296 households.  (3) Environmental vector management.  (4) Control group without interventions. | *Phlebotomus argentipes,* Phlebotomus spp., Sergentomyia spp.*, Leishmania donovani* |  | Baseline insecticide susceptibility tests:  The sand fly knockdown rate at 1 hour was 88%, and mortality at 24 hours was 100%. Of 9,766 sand flies trapped over the course of the intervention study, 8,295 (84.9%) were identified as *P. argentipes*, 900 (9.2%) as other *Phlebotomus spp*., and 571 (5.8%) as *Sergentomyia spp*.  In March and April 2007, IRS and ITNs were associated with statistically significant decreases in the density of both female and male *P. argentipes*.  There was no significant difference in *P. argentipes* density between the IRS and ITN arms at any time point from October 2006 through April 2007. In October 2007, *P. argentipes* density remained significantly lower in the ITN arm than in the control arm.  In October 2007, the density in the IRS arm was also significantly higher than in the ITN arm. The proportion of gravid female *P. argentipes* ranged from 28% to 40% in the baseline survey. | Both IRS and ITNs were associated with a 70–80% decrease in the density of *P. argentipes* 4 to 5 months after the intervention. ITN operations achieved significantly lower vector density compared with the control arm.  Both IRS and ITN show a potential role for VL vector control.  With ITNs, vector densities showed large and significant decreases compared with the control arm at every post-intervention sampling point.  They hypothesize that a more sustained impact may be achieved if IRS is used to effect a rapid decrease in sand fly populations, followed by wide distribution of ITNs to prevent high rates of leishmania transmission when the sand fly populations rebound. | There is a need to assess both entomological and disease control impacts. | 16/25 |
| Das et al., 2010, Nepal [4] | cRCT | Two districts in eastern part of Nepal (Sunsari and Morang): 24 clusters with 1335 HHs and 6955 habitants.  Baseline and follow-up: 2 weeks before and 2 weeks, 4 weeks and 5 months after intervention.  November 2006 to April 2007 | After stratification of villages on the basis of disease incidence, four groups of six clusters were randomly allocated to the four intervention arms: LLIN, IRS, EVM and Control  LLIN: sufficient PermaNet mosquito-nets were provided to cover every member of the household.  IRS: Synthetic pyrethroids; alpha-cypermethrin was used for spraying with a concentration of 25mg/m2.  EVM: lime was provided for wall repairing  Control: no interventions  Sand fly collection through light traps and mouth aspirators. | *Phlebotomus argentipes, P. papatasi, Sergentomyia spp.* |  | Baseline survey:  4661 sand flies were collected comprising 71% by light traps and 29% by mouth aspirators. *P. argentipes* occupied 49% of the total sand fly collection and remaining species were *P. papatasi* and *Sergentomyia* *spp*.  Follow-up surveys:  5280 sand flies were collected (60% by light traps and 40% by aspirators). *P. argentipes* occupied 26.6% of the total sand fly collection.  LLIN:  Vector density dropped from 7.9 to 0.9 per house per night by LT collections and from 1.8 to 0.5 per house per morning aspirator collections in three follow-up surveys after intervention.  IRS:  *P. argentipes* density decreased from 11.0 to 0.6 per house per night by LT collections and from 1.3 to 0.0 per house per morning by aspirator collections in the baseline to follow up surveys. IRS was highly effective for reducing vector density.  EVM:  *P. argentipes* density decreased from 8.2 to 2.6 per house per night in LT collections and from 0.6 to 0.1 per house per morning aspirator collections in the baseline to follow-up surveys. No significant difference in vector density while comparing with the control arm was observed. | LLINs may be more effective since deltamethrin has not only insecticidal effect but also repellent effect. Deltamethrin impregnated bed nets with fine mesh provided an effective barrier against vectors. LLINs seem to be a promising alternative to IRS for vector control.  IRS was the most effective tool for vector control. Besides lots of drawbacks during spraying and application procedures, it can be a good tool to reduce the sand fly density, if supervised properly.  EVM was found least effective as compared with LLIN and IRS in reducing the sand fly density. Sand fly density in this arm was found to be reduced for the first few months, however, it could not be decided whether it was due to weather or it was due to the chemical (CaCO3) in the lime. EVM could not represent itself as long-term vector control method, but it might be the alternative choice as a better non-insecticidal vector control method to be integrated with other intervention methods. | It was observed that the decline in sand fly density in all arms during first follow-up survey might be due to adverse environmental conditions as low temperature and humidity. During the subsequent follow-up surveys, increasing tendency in sand fly density might be the influence of favourable environmental condition and successive loss of effectiveness of the intervention methods. | 11/25 |
| Joshi et al., 2009, Bangladesh, India, Nepal [5] | cRCT | Study areas in Bangladesh, India and Nepal  24 clusters per study site with 120 HHs per site  Follow-up:  Collection of sand flies before the interventions and after 5 months in India and Nepal and after 6 months in Bangladesh. | Four study sites (6 clusters per intervention) with the following study groups:    IRS carried out by the district vector staff and the research team.  Deltamethrin in Bangladesh  DDT in India  Alpha cypermethrin in Nepal  LLIN: PermaNet nets with small mesh, polyester, resin coating containing deltamethrin distributed in all HHs.  EVM: In Nepal and India: wall plastering with a lime/mud mixture was promoted. In Bangladesh: plastering was done with mud only.  Control group: no intervention  Bioassays:  Determination of the efficacy of the insecticide sprayed surface and LLINs  Vector density in each cluster was monitored by CDC light traps during two consecutive nights, pre-intervention (November 2006) and post-intervention (April 2007). | *Phlebotomus argentipes,* *P. papatasi, Sergentomyia spp., Leishmania donovani* |  | The estimated intervention effect in terms of reduction in sand fly counts in the simple model showed a 72.4% reduction for IRS, a 42.0% reduction for EVM and a 43.7% reduction for LLIN.  IRS resulted in significant sand fly reductions in all sites independent of type of walls or dwelling or type of insecticide (DDT or pyrethroids).  LLINs had a significant negative effect on sand fly densities in India and Bangladesh, but not in the two Nepal sites.  EVM using mud for wall plastering in Bangladesh was not effective; EVM using lime plastering significantly reduced the sand fly densities in India and Nepal (Sarlahi and Sunsari districts) but not in the other Nepal site. | They showed that IRS, under quality controlled conditions and implemented by dedicated research teams, was efficacious in reducing the indoor VL vector density.  LLINs and EVM were also able to reduce VL vector densities at least for 5 or 6 months after the intervention, independent of housing conditions and the presence or absence of cattle.  LLINs had a significant effect on the number of sand flies in India and Bangladesh, but not in the two Nepali sites; when taking the sites together the intervention effect was also significant.  EVM using mud only for wall plastering does not seem to work.  IRS should be strengthened in India and Nepal. In Bangladesh, the insecticide treatment of existing bed nets could bring about an immediate reduction of vector populations.  In all three countries, operational research to inform policy makers about the efficacious options for VL vector control and program performance should be strengthened. | The site-specific data regarding LLINs and EVM have to be interpreted with caution because the limited number of clusters per arm provided uncertain estimates (wide confidence Intervals).  Interpreting the protective efficacy of LLINs, it has to be taken into consideration that CDC light trap captures monitor sand flies outside the bed nets, so that the actual protection for people sleeping under a LLIN from infective bites is most likely to be higher.  This study was conducted under fairly controlled conditions which are not easily applicable in a national vector control program. | 16/25 |

**S1 Table. Evidence table.** Continued

| **Author, year, country** | **Study type/ Methodology** | **Sample size/ Follow-up** | **Study arms /Interventions in detail** | **Species of Vector/ Species of parasite** | **Results:**  **Human outcome indicators** | **Results:**  **Entomological outcome indicators** | **Conclusions by the authors** | **Limitations described** | **Quality assessment scores by the authors** |
| --- | --- | --- | --- | --- | --- | --- | --- | --- | --- |
| **RCT** | | | | | | | | | |
| Banjara et al., 2019, Nepal [6] | RCT  (for the vector control part of the study) | Intervention activities were conducted in the Saptari district from June to August 2016 in VL-endemic villages.  264 intervention and 92 control HHs  Follow-up after 1, 3, 9 and 12 months | Combined camp approach:  Mobile teams conducted combined camps for fever and skin lesions to conduct VL/PKDL, malaria, tuberculosis, leprosy, and other febrile illnesses.  Incentive approach:  Female community health volunteers were trained to identify probable VL/PKDL cases, malaria, tuberculosis, and leprosy and to refer them to the hospital for confirmation and treatment.  Blanket HH screening and evaluation of interventions immediately after the fever camps.  Vector control interventions:  A RCT for vector control was performed to compare bednet impregnation with KOTAB123, wall painting with Inesfly 5AIGRNG™, IRS with deltamethrin, and a no-intervention control group.  Sandflies were collected during two consecutive nights with two CDC light traps per household by trained personnel.  The cone bioassay test was performed by using the World Health Organization Pesticide Evaluation Scheme (WHOPES) method at 1, 3, 9, and 12 months after the intervention.  Assessing the acceptability of vector control interventions by HH interviews. | *Phlebotomus*  *argentipes, Leishmania donovani* | Case detection:  The camps were attended by 398 people. Among the 398 people who attended the camp, 275 were further tested: 76 for suspected VL, 97 for tuberculosis, three for leprosy, and 99 for malaria. Of these, one each was positive for VL, leprosy, and malaria; and three were positive for tuberculosis.  In the incentive approach villages, no case was reported by female community health volunteers during the year following their training.  264 HH heads were interviewed for their perceptions and satisfaction about these vector control interventions: 94% perceived a reduction of sandflies after the application of insecticidal paint, 72% after bed net impregnation with insecticide, and 79% after IRS. | Reduction of the sandfly density up to 1 months after IRS application and bednet impregnation, whereas it was reduced for 12 months after insecticidal wall painting.  The bioassays performed on the treated surfaces showed that the mortality of *Phlebotomus argentipes* sandflies was about 95% at the 1-month follow-up and 80% at the 12-month follow-up on the painted surfaces; 50% and 26%, respectively, for insecticide-treated bednets; and 99% and 23%, respectively, for IRS. | Fever camps are effective in identifying active VL, tuberculosis, and malaria cases in the village, thus, achieving the dual objective of potentially reducing VL transmission and providing better care for other causes of febrile illness.  Insecticide wall painting provides long-lasting effects and is well accepted by the communities.  Insecticidal wall paint was effective for up to 12 months after intervention on sandfly density and mortality. By contrast, IRS, even in study conditions, was effective in the first month, but its efficacy waned thereafter both on density and mortality. Bednet impregnation with slow-release insecticide reduced density for the first month and had an effect on mortality for 9 months.  Insecticidal wall paint can be very valuable and sustainable for VL vector control as is effect lasts longer and its less technically challenging than IRS. It can also be combined with other vector control measures. | The combined fever camp approach would be more sustainable in a long term.  The incentive approach implemented through female community health volunteers did not identify any VL, PKDL, leprosy, malaria, and tuberculosis cases. By contrast, in Bihar, India, the accredited social health activists were able to refer 27% of the VL cases after one training session and 46% after two training sessions. This difference could be due to very low VL caseload in these villages in Nepal and the need for repeated training of the volunteers.  The characteristics of the villages were different. The villages with VL cases had also higher prevalence rates of other tropical diseases and fever in general.  The bednet impregnation intervention was carried out only in some target houses and the community effect was lost. | 15/25 |
| **Intervention studies** | | | | | | | | | |
| **Asia** | | | | | | | | | |
| Kumar et al. 2020, India [7] | Intervention study | Study site: Vaishali district of Bihar, India  Follow-up: from Jan 2016 to Dec 2016 | IRS village selection via GIS-based spatial mapping.  Integrated control strategy.  Two rounds of IRS were performed annually using 166 IRS squads.  In 2015, the first round of IRS was performed using DDT (WP 50% at a dosage of 1 g/m2). In the second round, DDT was used for the first 15 days then SP (alphacypermethrin 5% at 25 mg/m2).  Training, demonstration and human resource development: Training of doctors, supervisor, monitors and IRS operators.  Multi-level supervision and monitoring systems.  The creation of mass awareness and circulation of needful information was performed at zonal offices, schools, clubs, and community levels at different blocks. 8 endemic and 8 non-endemic villages were selected randomly for surveying the awareness.  Implementation and standardization of compression pump: using a modern hand compression pump.  Evaluation of DDT-IRS:  Insecticide Quantification Kit based IRS quality assessment  Susceptibility tests:  For local *P. argentipes* against DDT (at 4% dosage) and SP (at 0.05% dosage) 7 prior to each IRS round.  Pre- and post IRS sand fly density assessment:  With CDC light traps in human dwellings and animal shelters at 06:00 and 18:00.  A post-IRS (within 2 days) house-to-house field survey was conducted based on WHO guidelines for the assessment of IRS quality.  A door-to-door active case search was conducted in all endemic villages reported in the last year and in the new endemic villages reported in the implementation year. Two rounds (i.e. February-March and June-July adjusted with two annual peaks of VL cases in Vaishali) of the active case detection survey were conducted annually. | *Phlebotomus argentipes* | All 16 blocks of the Vaishali district achieved the VL elimination target in 2016. VL cases were reduced from 664 in 2014 to 163 in 2016 and endemic villages from 282 in 2014 to 142 in 2016.  The case reduction rate was increased from 22.6% in 2014 to 58.8% in 2016.  On average, 74 VL infected villages became Kala-azar free each year from 2015 to 2016. | The integrated VL vector control strategy is a useful tool for eliminating sand flies. | VL vector elimination is possible under strong monitoring and supervision.  Focal spraying is shown to be effective for interrupting intra-village transmission. The approach assumed that the instantaneous covering of all neighboring HHs through FS within 500 m of a new case location could kill all infected sand flies.  The success of a control program is directly related to the quality of the staff. Therefore, human resource development is necessary for all control programs.  The strategy presented in this study could be the stepping stone for achieving the VL elimination target in highly affected districts. | The study has not been used on a small scale but only during the attacking phase when the cases are widely distributed. Thus, reactive or proactive IRS might be cost effective during the sustenance phase when the cases are more localized and focalized, which has not been tested in the Vaishali study. | 13.5/18 |

**S1 Table. Evidence table.** Continued

| **Author, year, country** | **Study type/ Methodology** | **Sample size/ Follow-up** | **Study arms /Interventions in detail** | **Species of Vector/ Species of parasite** | **Results:**  **Human outcome indicators** | **Results:**  **Entomological outcome indicators** | **Conclusions by the authors** | **Limitations described** | **Quality assessment scores by the authors** |
| --- | --- | --- | --- | --- | --- | --- | --- | --- | --- |
| Mandal et al., 2019, India [8] | Intervention study | Study site: two villages of Mahnar block in Vaishali district, Bihar, India  Follow-up: from June 2015 to July 2016, 2 weeks prior to interventions and 2, 4 and 12 weeks after interventions | IRS using two insecticides [dichlorodiphenyltrichloroethane (DDT 50%) and synthetic pyrethroid (SP 5%)] was evaluated for VL-vector control.  Two rounds (first round, February–March; second round, June–July) of annual IRS during 2015 and 2016 were conducted by the State Health Society of Bihar  WHO cone-Bioassay Test:  Evaluation of the temporal residual efficacy of insecticides.  Tube-bioassay method:  Exploration of insecticide susceptibility.  Monitoring pre- and post IRS sand fly densities with CDC light traps in human dwellings and animal shelters between 18:00 and 06:00.  GIS-mapping  to map the household type distribution of insecticide susceptibility of the vector, and IRS-status of the households to interpret the spatiotemporal VL vector distributions | *Phlebotomus argentipes* | A total of 205 households were targeted for IRS in each round, of which 179 households (87.3%) in the DDT-round and 194 households (94.6%) in the SP-round accepted IRS for VL vector control. The percentage of fully sprayed households during SP-IRS (86.3%) was higher than the DDT-IRS (52.7%).  SP-IRS has been reported as having better community acceptance than DDT-IRS in all household types.  Reduction in *P. argentipes* counts due to SP-IRS was higher than DDT-IRS between household groups (i.e. sprayed and sentinel), in all intervals post-IRS. | *P. argentipes* sand flies were highly susceptible to alpha-cypermethrin (0.05%) but manifested a considerably lower susceptibility to DDT in the study villages.  Residual efficacies were varied between wall-surfaces; both insecticides failed to achieve the duration of IRS effectiveness recommended by the WHO. | The mean mortality rate of *P. argentipes* to DDT below 50% indicates a high-level resistance to DDT.  Housing characteristics, insecticide susceptibility of the vector and IRS-status combination could be useful in evaluating *P. argentipes* density.  GIS-based combined spatial-risk mapping (at a macro level) can be a useful tool for risk-zone identification for monitoring the emergence and resurgence of sand files in pre- and post-IRS sessions. | They did not evaluate the insecticides’ deposition on sprayed walls and the quality of insecticide used for IRS. Deviation in quality and quantity of the insecticides will affect the sand fly mortality rates and intervention effect of the IRS. Thus, estimated mortality rates between surface-types and the intervention effects between household groups may vary from the actual results. | 15.5/18 |
| Poché et al., 2018, India [9] | Intervention study | Study site: 24 villages in two Bihar districts: Suran and Muzaffarpur (India) | This study was part of a large-scale VL-incidence survey performed in 60 villages within each district (n = 120) in 2015.  Prior to study initiation, it was discovered that two rounds of IRS application had been performed within several villages in both districts in 2015 and that application would be repeated in 2016.  Biweekly collection of *P. argentipes* over 47 weeks in 12 villages of each districts with CDC light traps in cattle enclosures, houses, and outdoors in peri-domestic vegetation. | *Phlebotomus argentipes* |  | A total of 155,908 sand flies were captured, counted, and identified.  Vectors relative abundance was greatest from June to August when minimum temperatures were highest.  P. argentipes were most frequently collected from cattle enclosures. Many sand flies were found to have taken blood from multiple sources, with ~81% having blood fed on humans and ~60% blood feeding on bovines. Differences in VL vector between IRS-treated and untreated villages in only ~9% of evaluation periods occurring during the peak period of human-vector exposure (June-August) and in ~8% of the total observations.  If P. argentipes abundance was decreased in IRS-treated villages, the decrease was only temporary.  Results suggest IRS-treatment has a limited impact on vector density. | Vector abundance has not significantly declined in IRS-treated villages.  Results suggest P. argentipes to 1) feed opportunistically on humans and bovines, 2) show a preference for cattle enclosures, and 3) be present outdoors in village vegetation. | These results are limited temporally, given that post-treatment collections were performed only twice in April, during which results of the current study and those of previous researchers suggest sand fly abundance in Bihar declines naturally. | 13.5/18 |
| Chowdhury et al, 2018, Bangladesh [10] | Intervention study | Selection of 8 highly endemic upazilas in Mymensingh district in 2012 pre-monsoon (May-June). The study was carried out from March to October 2012.  Observation of spraying squads: 136 squads in 5 upazilas.  HH acceptability survey: 600 HH.  Vector density measurement: 36 HH from each treatment group.  Bioassay on sprayed surfaces: 40 HH in each treatment group.  Measurement 2 weeks prior IRS.  Follow-up after 1 and 5 months. | Observation of IRS activities using check lists and questionnaires included in the WHO/TDR monitoring and evaluation tool kit by trained research assistants.  Community satisfaction survey one-month post-spraying: HH acceptability survey of IRS was conducted in all study areas using a structured questionnaire in 600 HHs.  Monitoring of vector density using CDC light traps in one upazila. Sand fly density was measured 2 weeks prior to IRS, at one and five months post-IRS in 36 sprayed and 36 unsprayed (control) HHs.  Bioassays:  Bioassays, using the WHO cone-method, were carried out in 80 HHs (40 sprayed and 40 unsprayed) to measure the effectiveness of the insecticide on sprayed surfaces. | *Phlebotomus argentipes* | Observation of IRS operations of 136 squads (544 spraymen and 136 squad leaders), in five endemic upazilas. Of the 544 spraymen interviewed pre-IRS, 60%, 3% and 37% had received training for one, two and three days respectively. During spraying activities, 64% of the spraying squads had a supervisor in 4 upazilas but only one upazila (Mukthagacha) achieved 100% supervision of squads. Overall, 72.8% of the spraying squads in the study upazilas had informed HHs members to prepare their houses prior to spraying.  All the respondents in the 600 HHs of the five study upazilas mentioned that their houses had been sprayed with insecticide and 94% said that their living rooms and cattle sheds had also been sprayed. Only 36.2% of interviewers had been informed about IRS in advance. 85.3% of the respondents said that they were happy with the IRS activities. | A total of 4132 sand flies were trapped at baseline, 1 month and 5 months post-intervention, of which 80.1% were P. argentipes.  At baseline, the number of P. argentipes was significantly lower in the IRS treatment group compared with the control group in both male and female, only female, and gravid female whereas no significance difference was observed in only male. Similarly, at one month post-IRS, there was a significant difference in P. argentipes densities between the treatment groups for both male and female and for only male. But no significant difference was found for only female and gravid female. At 5 months post-IRS, no significant difference in P. argentipes densities was observed between treatment groups for both male and female, for only male, for only female and for gravid female.  One month after IRS the P. argentipes density was reduced by 22.61% and 118.79% in both male and female, and only male which dropped to 6.37% and 53.94% at five months respectively. However, no reduction was found in only female and gravid female P. argentipes density. | IRS is able to control the increase of only male P. argentipes density in the IRS areas up to one month post spraying. But it fails to control the P. argentipes density in IRS houses at five months.  Bioassay showed that the insecticidal effect rapidly disappeared.  The communities positively accepted the IRS operations as they had not experienced any IRS or other vector control activities for a long time.  IRS is highly effective in quickly reducing vector densities underlining the need to continue the operations.  IRS is an effective but also expensive vector control tool. Therefore, it is recommended that the national programme ensure: (1) procurement of quality insecticide, (2) proper training of human resources involved in IRS operation, (3) proper monitoring and supervision during spraying, (4) regular vector surveillance and bioassays on sprayed surfaces, (5) routine testing of vector susceptibility and (6) community sensitization. | The overall performance of house spraying was poor. Issues with the late release of fund, low daily wage of spraymen and squad leaders, and local pressure are hindering the timely recruitment of spraymen and squad leaders.  More than 50% spraying squads found that there was no one home while they were in the village. This points to a communication gap between the community and the service provider.  They found poor compliance with the guidelines, apart from mentioning the team number.  They had to comply with the micro action plan made by the sub-district managers for all monitoring visits as they monitored their IRS activities. In such a case, there could have been a chance of leakage about the monitoring visits to IRS squads from beforehand. To avoid such circumstances, we did not disclose our detailed plan of visits to local health authorities so that the quality and validity of data were ensured. | 14.5/18 |

**S1 Table. Evidence table.** Continued

| **Author, year, country** | **Study type/ Methodology** | **Sample size/ Follow-up** | **Study arms /Interventions in detail** | **Species of Vector/ Species of parasite** | **Results:**  **Human outcome indicators** | **Results:**  **Entomological outcome indicators** | **Conclusions by the authors** | **Limitations described** | **Quality assessment scores by the authors** |
| --- | --- | --- | --- | --- | --- | --- | --- | --- | --- |
| Kumar et al., 2017, India [11] | Intervention study | Study site:  Samastipur district, Bihar, India  400 HHs were included.  Follow-up: from Oct 2014 to Oct 2015 | 4 interventions arms: IRS in Mirzapur, ITN in Sahnitola, IRS+ITN in Nifsy, Control (no chemical treatment) in Bisanpur  ITNs (Perma Net 3.0) were distributed at the Intervention sites of ITN and IRS+ITN during the month of October.  IRS was done in each household of village Mirzapur and Nifsy allocated with IRS and IRS+ITN respectively (suspension of 5% DDT).  Baseline interview survey: upon sociodemographic information and sand fly density observation 7 days prior to the intervention.  Sand fly collections:  with aspiration technique (AT) and light trap technique (LTT).  The residual activity of IRS and ITN was measured at each interval of 3 months for  estimating the Bioavailability of Insecticide in Intervention (BII) | *Phlebotomus argentipes, Leishmania donovani* | 255 and 270 ITNs were distributed in the villages for ITN (Sahnitola) and IRS+ITN (Nifsy) intervention respectively so as to cover entire sleeping spaces of population at these villages. The population-treated net distribution ratio was stabilized as 3:1.  Socio-demographic observation revealed that majority of houses at the study were of thatched and mud plastered type, serving as best habitat and favorable conditions for surviving sand flies as well as disease transmission among human hosts.  Among the study site, availability and uses of bed nets by the households before the intervention session were observed as 22.14%, 26.17%, 24.16%, 27.51% at Bisanpur, Sahnitola, Mirzapur, and Nifsy; for intervention as control, IRS, ITN, IRS+ITN respectively.  All respondents facilitated with ITNs, confirmed the proper usage of provided bed nets as well as the continuous good physical conditions of the nets. Almost the only reported side effect was unpleasant smell, particularly in the 2 arms that included IRS and IRS+ITN. The perception of added benefits (mainly reduction in nuisance of insects) was highest in villages where ITNs were involved.  Overall satisfaction was achieved in the villages involving ITNs as compared to the village with IRS as single intervention with 87% acceptability. | Of the total number of 679 *P. argentipes*, collected during 12 months from all sites, 33.43% and 66.56% were collected with the AT and LTT respectively. However, the highest collection of unfed female sand flies (74.13%) was recorded with the nocturnal LTT as compared to the diurnal AT (13.04%). In contrast, the highest percentage of fed and gravid female sand flies (44.22% and 42.75% respectively) were recorded with the AT as compared to those collected with LTT.  Out of all 679 sand flies collected from the intervention sites, 77 were fed female sand flies. Only 2 fed females were caught using AT from the village with the combined intervention (IRS and ITN) while the rest (75 fed female sand flies) were collected from the other villages including either of single interventions or control. Likewise, only 6 gravid sand flies were collected in the combined intervention village and the other 97 gravid sand flies were collected from the villages with either ITN or IRS interventions.  The lowest numbers/proportions of sand flies were collected from villages with the combined approach (IRS plus ITN) as compared to single intervention sites (either IRS or ITN only) or the control site.  The reduction of insecticidal content of IRS was faster and more pronounced (exhibiting corrected mortality rate as 52.38%, 58.33%, 45.45% & 50.00%) as compared to ITN (with corrected mortality rate as 84.44%, 82.50%, 77.78% & 83.33%) over the period of 13 months since intervention.  The monthly observation of percent reduction (% RI) of sand fly density due to intervention establishes the highest % RI (93.59-100%) at the sites with IRS+ITN as compared to either at the control site (with 0% reduction) or with single intervention of IRS (with 4.29-86.77%) or with ITN (60.18- 97.01%).  At the site with the combined treatment of IRS+ITN, no re-emergence of sand flies was recorded till 13 months following the intervention. | Regarding sandfly collection techniques, it can be inferred that both collection techniques applied in the study complement each other for trapping both types of sand flies depending upon their nature and it is beneficial to use them together.  The efficacy of IRS and ITN when applied alone, gets depleted over the period of time producing unsatisfactory result for driving VL vector population as well as competing the instances of increased VL cases. Whereas combined strategy provides additional protection for combating the insects’ menace as well as for driving VL transmission at a lowest level.  The additional benefits of IRS plus ITN perceived by the population was observed to be so advantageous and eco-friendly that it nullified the negative impact of interventions and hence resulted into absolute satisfaction and acceptability for interventions.  The limitations with IRS and ITNs as single interventions can be overcome by a combined approach that should be rigorously collected through randomized controlled trials. | The expected outcome at the villages of Sahnitola (for intervention with ITN only) was insignificantly hampered due to poor literacy rate i.e., 28% only. | 13/18 |
| Coleman et al., 2015, India [12] | Intervention study | 8 VL endemic districts in Bihar State  Pre-IRS surveys were performed in three districts, and post-IRS surveys were performed in 8 districts. | Quality assurance of IRS:  Was performed in 8 VL endemic districts that were scheduled to receive IRS in 2014.  Implementation of a modified version of the WHO TDR Monitoring and Evaluation Toolkit for IRS study design  Pre-IRS quality assurance surveys to determine the level of residual DDT in houses in three districts were conducted in February 2014. Entomology study sites and sample collection: Protocols for measuring impact on vector densities and insecticide susceptibility from the WHO/TDR Monitoring and Evaluation Toolkit were used. Data were collected in February 2014 (pre-IRS), 1 moth post-IRS (May–July 2014), and 3 months post-IRS (July–September 2014).  Abundance data were collected using CDC light traps set up in six randomly selected houses over a period of one night (6:00 PM to 6:00 AM).  Sample collection:  Samples to determine the concentration of DDT delivered to walls during IRS were collected from wall surfaces using 5-cm^2^ Bostik tape discs.  Susceptibility assays:  Sand flies were exposed to DDT (4%) or deltamethrin (0.05%) WHO-impregnated papers. | *Phlebotomus argentipes* |  | Pre-IRS surveys:  360 IRS wall samples from 90 HHs were tested for DDT residue concentration.  91.3% of the samples had DDT concentrations below 0.1 g ai/m^2^.  Post IRS-surveys:  Samples were collected in 560 HHs between 0 and 45 d post-spraying.  The average level of DDT found on walls post-IRS was 0.37 g ai/m^2^  Matched pre-IRS and post-IRS samples were compared using the Wilcoxon signed rank test (matched for household, wall position, and surface type). There was a 10-fold difference between the mean pre-IRS and post-IRS concentrations of DDT, although the concentrations of DDT were still well below the target dose post-IRS.  Susceptibility tests with wild-caught P. argentipes sand flies against 4% DDT WHO- impregnated papers in non-IRS and IRS villages showed corrected mortality ranges of 24.6–37.5% in control villages and 30.0–37.8% in IRS villages across the four districts sampled.  In IRS villages, a total of 433 sand flies were caught in February 2014, whereas in non-IRS villages, a total of 640 sand flies were collected. | An effective insecticide needs to be sprayed at a biologically effective concentration on the right structures at the right time.  Good IRS programs should have an appropriate monitoring and evaluation system in place that allows evidence-based insecticide choice and quality assurance of the implementation of the spray program  The effectiveness of the IRS program is dependent on the quality of the insecticide, formulation, and spray activities combined with the susceptibility status of the local sand fly populations. | Pre-IRS survey results showed that after a median time of 3.5 months, 91.3% of walls had a residual DDT level of less than 0.1 g ai/m^2^. This finding could be due to rapid degradation of the insecticide, quicker absorption into the walls than is commonly assumed, or low target concentrations being delivered to walls.  No diagnostic dose has been determined directly for sand flies for DDT and deltamethrin insecticide susceptibility testing. | 12.5/18 |

**S1 Table. Evidence table.** Continued

| **Author, year, country** | **Study type/ Methodology** | **Sample size/ Follow-up** | **Study arms /Interventions in detail** | **Species of Vector/ Species of parasite** | **Results:**  **Human outcome indicators** | **Results:**  **Entomological outcome indicators** | **Conclusions by the authors** | **Limitations described** | **Quality assessment scores by the authors** |
| --- | --- | --- | --- | --- | --- | --- | --- | --- | --- |
| Chowdhury et al, 2011/2, Bangladesh [13] | Intervention study | Districts of Vaishali with 116.056 HHs (in the Indian state of Bihar), Sarlahi with 111.076 HHs (in Nepal) and Sunsari with 5153 HHs (also in Nepal).  The study was carried out from March 2008 to March 2009. | The study villages were sprayed, by the national VL-control programmes, in April–June 2008.  Six methodological tools were used to measure the performance and results of the IRS:  (1) Formal interviews using a structured questionnaire with the district officers responsible for the VL- control programme.  (2) Structured observations of the spraying teams, with a standardized checklist used.  (3) The monitoring of *P. argentipes* densities at pre- intervention and 2 weeks, 4 weeks and 5– 6 months after IRS, to assess ‘effectiveness’.  (4) The bio-assay-based monitoring of the bio- availability of insecticides on sprayed sur-faces  (5) The quantification of insecticide concentrations on sprayed walls.  (6) The assessment, in tube bio-assays with standard treated papers, of the susceptibility of local *P. argentipes* to the insecticide used in the national IRS in India [i.e. 1, 1,1-trichloro-2,2-di(4-chlorophenyl)ethane (DDT)] or to a pyrethroid (deltamethrin) similar to the lambda-cyhalothrin used in Nepal.  The bio-assays were performed three times in each test house: 2 weeks, 4 weeks and 5–6 months after the IRS. | *Phlebotomus*  *argentipes* | IRS programme managers used the relevant technical guidelines and were familiar with the procedures for IRS. | The results of bio-assays and the chemical analysis of samples from sprayed walls indicated substandard spraying and suboptimal concentrations of insecticide on sprayed surfaces.  Sandfly resistance to DDT in India was widespread but the potential vectors in Nepal remained very susceptible towards a pyrethroid (deltamethrin) similar to the lambda-cyhalothrin. | The substantial and sustainable reduction of VL vectors, to interrupt the transmission of *L. donovani*, seems unlikely.  Preparation and monitoring of IRS operations against sandfly populations in India and Nepal need to be improved. | There are significant operational challenges, and this was particularly obvious in Nepal’s Sunsari district, where the management of pressure pumps and spraying performance was poor, only a small fraction of the target insecticide concentration was found on sprayed sur- faces, and, in consequence, only a small and short-lived reduction in *P. argentipes* densities was measured.  The 5 months follow-up was probably made invalid by flooding or lime plastering in the study areas. | 10.5/18 |
| Kumar et al., 2009, India [14] | Intervention study  Before and after, no control intervention study | Study site: 119 endemic villages in Vaishali, Muzaffarpur, East Champaran and Saran districts  Baseline: Cases reported in Dec 2006  Follow-up: Evaluation of sand fly density prior to DDT spraying, one month and three months post spraying. | Extensive DDT spraying program by the Bihar Government from 15 Feb 2007 to 15 April 2007.  Monitoring of spraying operations and random house visits.  Evaluation of sand fly densities by standard aspirator method with the help of three-celled torch.  500 houses were interviewed randomly in all the study districts regarding social acceptability and perceptions of the spray operation. | *Phlebotomus argentipes* | In most of the districts, the incidence of kala-azar cases reduced after the DDT spray coupled with IEC activities and government efforts.  There are few endemic districts like Saharsa, Saran, Muzaffarpur and Vaishali districts that have shown increase in the incidence of cases in the month of December 2007 in comparison with December 2006.  Community acceptance: out of the 500 households examined, 4.8% has totally refused the spraying due to different reasons and 9.6% refused partially. | After spraying the house index of *P. argentipes* de-  creased considerably when compared to pre-spray data in the study districts.  Significant difference in the density of sandflies during pre- and post-spray periods in Vaishali, Saran and East Champaran districts and there was no significant difference in Muzaffarpur district. | DDT is effective in controlling sand fly populations.  It is suggested to strengthen the IEC activities to sensitize the community and thorough monitoring of spraying operation is essential to achieve the desired result in stipulated time. With proper monitoring and training, two rounds of DDT spray with good house coverage in all the endemic districts up to three years and proper treatment of cases are essential for total elimination of kala-azar in Bihar state. |  | 11/18 |
| Mukhopadhyay et al., 1996, India [15] | Intervention study | Study site: three villages, Dariasudhi in North 24-Pargana district, and Chatrapara and Mulchatki in South 24-Pargana district, West Bengal, India  Follow-up: sand fly collection after DDT spray every 2^nd^ or 3^rd^ week from Jan to Dec 1994 | Hamlet 1 in Dariasudhi: 26 Kala-azar cases in 1992-1993  Hamlet 2 in Dariasudhi: no Kala-azar cases  Chatrapara: Two Kala-azar cases and 7 PKDL cases in 1993  Mulchatki: no Kala-azar cases  Intervention areas: Hamlet 1 of Dariasudi and Chatrapara  Control areas: Hamlet 2 of Dariasudi and Mulchatki  Sand fly collection: early in the morning from different indoor habitats using standard mouth suction aspiration and torch light.  IRS with DDT at the rate of 1g/m^2^ in intervention villages in Dec 1993.  Susceptibility tests using 4% DDT according to WHO standard technique. | *Phlebotomus argentipes, P. papatasi* |  | The density of *P. argentipes* was reduced to almost zero level in all biotopes up to Sep 1994 in interventions areas. No drastic reduction in vector densities was noted in the control areas.  Susceptibility status of *P. argentipes* to 4% DDT shows a 100% mortality of the flies in 30 and 60min exposure, and 55% mortality in 15min exposure.  *P. papatasi* was found in low densitiy in all study sites throughout the hole period.  Susceptibility status of *P. papatasi* to 4% DDT shows a 20% mortality of the flies in 60min exposure, and 96% mortality in 120min exposure. | *P. argentipes* and *P. papatasi* are susceptible to DDT in West Bengal. |  | 5.5/18 |
| Kaul et al., 1994, India [16] | Intervention study | Study sites: two contiguous villages, Shujabad (ca. 5000 people) and Bhojpura (ca. 3600 people), situated on the banks of the Ganges  Kala-azar and PKDL cases were found in Shujabad in 1990 and 1991. No cases were reported for Bhojpura.  Intervention: June and August in 1991  Sand fly collections: 18.-21. Sep. | Intervention site: Shujabad: All human dwelling and cattlesheds were sprayed with DDT (dose1g/m2) by district health authorities. The spraying was limited to about 1.7m from the inner walls of premises.  Sand flies were collected during a period when peak population densities of the vector species are encountered.  Clusters of dwellings in both, eat-west and north-south directions were chosen for sand fly collections (Shujabad: 71 dwellings, Bhojpura: 55 dwellings).  Sand fly collections with suction tubes from dark corner, ceilings and crevices. Sometimes they were driven out of the catch with the help of cigarette smoke. | *Phlebotomus argentipes, P. papatasi, Sergentomyia spp.* |  | Bhojpura: collection of 365 sand flies (91.7% *P. argentipes*).  Shujabad: collection of 14 sand flies (0% *P. argentipes* and *P. papatasi*). | The population of *P. argentipes* was highly susceptible to DDT. Therefore, IRS was highly effective and prevented the build-up of the vector population during transmission season. |  | 5.5/18 |

**S1 Table. Evidence table.** Continued

| **Author, year, country** | **Study type/ Methodology** | **Sample size/ Follow-up** | **Study arms /Interventions in detail** | **Species of Vector/ Species of parasite** | **Results:**  **Human outcome indicators** | **Results:**  **Entomological outcome indicators** | **Conclusions by the authors** | **Limitations described** | **Quality assessment scores by the authors** |
| --- | --- | --- | --- | --- | --- | --- | --- | --- | --- |

| **South America** | | | | | | | | | |  |
| --- | --- | --- | --- | --- | --- | --- | --- | --- | --- | --- |
| Barata et al., 2011, Brazil [17] | Intervention study  Before and after, no control | Study site:  municipality of Montes Claros, in northern Minas Gerais State, Brazil  Follow-up: from September 2005 to August 2006, monthly sand fly captures in 10 districts of Montes Claros | Sand fly captures with CDC light traps: Two traps were placed in one residence per district for three consecutive days a month, one inside the house and another in the peridomicile, totaling 20 traps.  Insecticide spraying with cypermethrin pyrethroid (125mg/m^2^), according to Brazilian Ministry of Health recommendations, was carried out in two cycles: November 2005 and May 2006. The insecticide was applied to internal and external walls of the 10 selected houses and their annexes (chicken coops, stables and warehouses) and in all residence in the neighborhood. | *Lutzomyia* spp. |  | 85.8% of sand flies were captures outside the houses. The sand fly fauna comprised 10 different species. *L. longipalpis*, *L. sallesi* and *L. intermedia* constitute approximately 90% of the fauna captured. These species were sensitive to treatment with the insecticide.  The two months prior to each spraying campaign (Sep-Oct 2005 and Mar-Apr 2006) were compared with the subsequent marking periods. The results showed that, two months after spraying, significant reduction occurred only outdoors. In the second spraying period, the differences between pre- and post-spraying were significant at two months and four months after spraying. Thus, the insecticide was less effective in the first cycle (two months) and more effective in the second cycle (four months). | Clear predominance of *L. longipalpis* (79%) both inside and outside domiciles.  The authors suggest that this species has been frequently found in the home environment, and is perhaps better adapted to the presence of man and domestic animals in endemic areas of visceral leishmaniasis.  The number of captured insects dropped abruptly immediately after application of two cycles of insecticide, i.e., November 2005 and May 2006, suggesting temporary efficacy in reducing sand fly population density.  The authors recommend three or four insecticide sprayings every year to achieve effective control of the sand fly population. |  | 9.5/18 | |
| **Survey** | | | | | | | | | |  |
| Hasker et al., 2012, India [18] | Survey in existing cohort study | Study site: 50 villages (200 hamlets) in the Muzaffarpur District of Bihar, India  Follow-up: three annual surveys in Sep and Oct of 2008, 2009 and 2010. | In each survey, they visited all HHs and collected demographic information. Additionally, they asked whether the house had been covered by indoor residual insecticide spraying in the year preceding the survey. In each survey, they also collected information about VL in the household since the previous survey. For the first survey, they used a recall period of 1.5 years.  At the time of the second survey in 2009, we also collected information about assets owned by each household, including domestic animals, and we recorded characteristics of the structure of the house and the surrounding vegetation. | *Phlebotomus argentipes* | Study population of 81.210 persons, divided over 13.416 HHs.  During the study period, we registered 207 VL cases, equivalent to an average annual incidence of 72.8/100,000 population.  VL was strongly associated with age; the odds of having VL was lowest for children <5 years of age and highest for children 5–14 years of age (odds ratio [OR] 2.5, 95% CI 1.5–4.0).  Higher socioeconomic status was associated with reduced risk.  IRS coverage was poor. In 2009 (the last year for which data were collected for the full year), only 12% of all households had reportedly been sprayed at least once. | Ownership of goats and presence of bamboo trees near the house are risk factors, but are not strong enough to warrant specific interventions.  Poor housing is a stronger risk factor; thus, housing plans launched by the Indian government may positively affect control of VL. | The Musahars are known to be among the poorest of the poor, but even after we controlled for confounding by socioeconomic status, the association remained statistically significant. Some residual confounding cannot be ruled out, but other factors probably play a role. One such factor could be long delays in seeking health care by Musahars, which was documented in another recent study |  | 11/18 | |
| **Modelling studies** | | | | | | | | | |  |
| Gupta et al., 2020, India [19] | Modelling study | Study site: 33 out of 38 districts of Bihar, India  Follow-up: Jan 2012 to Dec 2017 | The authors compared the rate of incidence decrease in Vaishali to other districts in Bihar via an interrupted time series analysis with a spatiotemporal model, and estimated the number of cases averted by the pilot. |  | Changes in Vaishali's rank among Bihar's endemic districts in terms of monthly case numbers showed a change pre-pilot (3rd highest out of 33 reporting districts) versus during the pilot (9th). The rate of decline in Vaishali's cases was 26^th^ highest pre-pilot and 19th during the pilot. Model simulations suggest a median 1,071 cases were averted in Vaishali between March 2015-December 2017. |  | Existing interventions when applied in combination and with special attention to quality could significantly reduce incidence.  Strengthening control strategies may have precipitated a faster decline in VL case numbers in Vaishali and suggests this approach should be piloted in other highly endemic districts. | The authors cannot conclusively attribute the additional decline in case numbers in Vaishali from 2015 to the intensified control program because this is an observational study.  They do not know the treatment information of some Vaishali cases that migrated to nearby district hospitals nor other districts’ cases migrating into Vaishali. It is also unclear how drug supply may have impacted incidence since the national program introduced single-dose liposomal amphotericin B in 2015-2016. | 13/18 | |
| Hasker et al., 2018, India [20] | Modelling study | Study site: the Muzaffarpur Health and Demographic Surveillance Site, a rural area of Muzaffarpur district, Bihar, India  Data of 14.376 HHs with 91.908 persons  Follow-up: from 2007 to 2015 | Establishment of optimal target areas for IRS an (re)active case finding.  They plotted incident VL cases on a map within a 6-months period (Jan to June or July to Dec). Buffers of 0, 50, 75, 100, 200, 300, 400 and 500 m around these cases were drawn.  Recording of total population and VL case numbers diagnosed over the period in each of these buffers and beyond. Incidence rate ratios were calculated using the population at more than 500m from any case as reference category. | *Phlebotomus*  *Argentipes, Leishmania donovani* | The risk of being diagnosed with VL within the next 6-month period was on average 45.2, 15.4, 14.6, 13.4, 9.2, 7.1, 5.9, and 5.1 times higher for those living in the same household or within 50, 75, 100, 200, 300, 400, or 500 m, respectively.  There was a very strong degree of spatial clustering of VL with incidence rate ratios ranging from 45.2 for those living in the same households to 14.6 for those living within 75 m of a case diagnosed, during the previous period. Up to 500 m the incidence rate ratio was still five times higher than that of the reference category. |  | The findings indicate that it is important to screen also HHs within a perimeter of 50-75m from an index case.  Further clustering occurs at immediate neighborhood and HH level.  Covering a perimeter of 500m with IRS, seems to be a rational choice. Therefore, control interventions should also target the close surroundings of reported VL cases.  Even effective IRS within a specific buffer zone would not be sufficient to prevent all cases in the next 6-month period for several reasons. First of all IRS would not prevent VL in a person already infected but still in the incubation phase, secondly because people may also be infected in outdoor locations. |  | 13/18 | |
| Stauch et al., 2014, India [21] | Modelling study | 1970 to 1986 | Usage of a previously published VL model that has been used to investigate emerging resistance against antimonial treatment.  Development of a system of ordinary differential equations to model the transmission dynamics of L. donovani between sand flies and humans on the Indian subcontinent.  Investigation of transmission thresholds dependent on measures reducing the sand fly density either by killing sand flies (e.g., indoor residual spraying and long-lasting insecticidal nets) or by destroying breeding sites (e.g., environmental management). | *Leishmania donovani* | The elimination of VL is possible if the sand fly density can be reduced by 67% through killing sand flies, or if the number of breeding sites can be reduced by more than 79% through environmental management. | Treated nets and to a minor extent IRS, predominantly kill sand flies that are about to transmit the infection, whereas breeding site control generally reduces the number of flies, regardless of whether they reach an age where transmission occurs. | Reduction of the vector's life expectancy is more effective than a reduction of the vector's breeding site capacity.  LLIN are a highly effective intervention tool because treated nets can be considered as baited traps that kill predominantly sand flies that are about to transmit the infection.  Three major reasons may limit the effectiveness of LLIN: (1) inappropriate usage of LLIN by man, (2) changed and/or alternative feeding or resting behaviour of the vectors and (3) vector adaptation or habituation against insecticidal substances.  Destroying breeding sites of P. argentipes is a promising tool for intervention and should also prevent re-emergence of infection after local extinction.  Resistance against DDT continues to spread and cross-resistance may emerge (e.g., in anophelines, the so-called knockdown resistance, a DDT/pyrethroid cross-resistance, is commonly found. Thus, IRS may only be a transient measure to effectively reduce sand fly density.  Integrated vector management, which combines different vector control measures, could be an effective approach to overcome the limitations of independently applied vector control strategies. | Uncertainty in parameter estimation translates into uncertainty of R_e_. Additional variability can emerge when further parameters such as temporal or spatial heterogeneities, which were not considered in our approach, are included.  The model was calibrated to average prevalence under the assumption of homogeneous spread of the infection among humans and vectors.  Animals serving as alternative blood hosts were not considered.  The infection probabilities for susceptible flies when feeding on a VL- or PKDL-patient and for susceptible humans after being the blood meal of an infected sand fly are assumed to be 100%. | 13/18 | |

**S1 Table. Evidence table.** Continued

Reference

1. Huda MM, Ghosh D, Alim A, Almahmud M, Olliaro PL, Matlashewski G et al. Intervention Packages for Early Visceral Leishmaniasis Case Detection and Sandfly Control in Bangladesh: A Comparative Analysis. Am J Trop Med Hyg 2019; 100(1):97–107.

2. Chowdhury R, Faria S, Huda MM, Chowdhury V, Maheswary NP, Mondal D et al. Control of Phlebotomus argentipes (Diptera: Psychodidae) sand fly in Bangladesh: A cluster randomized controlled trial. PLoS Negl Trop Dis 2017; 11(9):e0005890.

3. Chowdhury R, Dotson E, Blackstock AJ, McClintock S, Maheswary NP, Faria S et al. Comparison of insecticide-treated nets and indoor residual spraying to control the vector of visceral leishmaniasis in Mymensingh District, Bangladesh. Am J Trop Med Hyg 2011; 84(5):662–7.

4. Das ML, Roy L, Rijal S, Paudel IS, Picado A, Kroeger A et al. Comparative study of kala-azar vector control measures in eastern Nepal. Acta Trop 2010; 113(2):162–6.

5. Joshi AB, Das ML, Akhter S, Chowdhury R, Mondal D, Kumar V et al. Chemical and environmental vector control as a contribution to the elimination of visceral leishmaniasis on the Indian subcontinent: cluster randomized controlled trials in Bangladesh, India and Nepal. BMC Med 2009; 7:54.

6. Banjara MR, Das ML, Gurung CK, Singh VK, Joshi AB, Matlashewski G et al. Integrating Case Detection of Visceral Leishmaniasis and Other Febrile Illness with Vector Control in the Post-Elimination Phase in Nepal. Am J Trop Med Hyg 2019; 100(1):108–14.

7. Kumar V, Mandal R, Das S, Kesari S, Dinesh DS, Pandey K et al. Kala-azar elimination in a highly-endemic district of Bihar, India: A success story. PLoS Negl Trop Dis 2020; 14(5):e0008254.

8. Mandal R, Kumar V, Kesari S, Das P. Assessing the combined effects of household type and insecticide effectiveness for kala-azar vector control using indoor residual spraying: a case study from North Bihar, India. Parasit Vectors 2019; 12(1):409.

9. Poché DM, Garlapati RB, Mukherjee S, Torres-Poché Z, Hasker E, Rahman T et al. Bionomics of Phlebotomus argentipes in villages in Bihar, India with insights into efficacy of IRS-based control measures. PLoS Negl Trop Dis 2018; 12(1):e0006168.

10. Chowdhury R, Chowdhury V, Faria S, Islam S, Maheswary NP, Akhter S et al. Indoor residual spraying for kala-azar vector control in Bangladesh: A continuing challenge. PLoS Negl Trop Dis 2018; 12(10):e0006846.

11. Kumar V, Rama A, Mishra P, Siddiqui N, Singh R, Dasgupta R et al. Investigating Associative Impact of Indoor Residual Spray and Insecticide Treated Nets for Minimizing Visceral Leishmaniasis Vector Population in Bihar (India). IJTDH 2017; 23(4):1–15.

12. Coleman M, Foster GM, Deb R, Pratap Singh R, Ismail HM, Shivam P et al. DDT-based indoor residual spraying suboptimal for visceral leishmaniasis elimination in India. Proc Natl Acad Sci U S A 2015; 112(28):8573–8.

13. Chowdhury R, Huda MM, Kumar V, Das P, Joshi AB, Banjara MR et al. The Indian and Nepalese programmes of indoor residual spraying for the elimination of visceral leishmaniasis: performance and effectiveness. Ann Trop Med Parasitol 2011; 105(1):31–5.

14. Kumar V, Kesari S, Dinesh DS, Tiwari AK, Kumar AJ, Kumar R et al. A report on the indoor residual spraying (IRS) in the control of Phlebotomus argentipes, the vector of visceral leishmaniasis in Bihar (India): an initiative towards total elimination targeting 2015 (Series-1). J Vector Borne Dis 2009; 46(3):225–9.

15. Mukhopadhyay AK, Hati AK, Chakraborty S, Saxena NB. Effect of DDT on Phlebotomus sandflies in Kala-Azar endemic foci in West Bengal. J Commun Dis 1996; 28(3):171–5.

16. Kaul SM, Sharma RS, Dey KP, Rai RN, Verghese T. Impact of DDT indoor residual spraying on Phlebotomus argentipes in a kala-azar endemic village in eastern Uttar Pradesh. Bull World Health Organ 1994; 72(1):79–81.

17. Barata RA, Michalsky EM, Fujiwara RT, França-Silva JC, Rocha MF, Dias ES. Assessment of sand fly (Diptera, Psychodidae) control using cypermethrin in an endemic area for visceral leishmaniasis, Montes Claros, Minas Gerais State, Brazil. Cad Saude Publica 2011; 27(11):2117–23.

18. Hasker E, Singh SP, Malaviya P, Picado A, Gidwani K, Singh RP et al. Visceral leishmaniasis in rural bihar, India. Emerg Infect Dis 2012; 18(10):1662–4.

19. Das P, Gupta VK, Siddiqui NA, Pollington TM, Mandal R, Das S et al. Impact of Intensified Control Strategies on Incidence of Visceral Leishmaniasis in a Highly Endemic District of Bihar, India. SSRN Journal 2020.

20. Hasker E, Malaviya P, Cloots K, Picado A, Singh OP, Kansal S et al. Visceral Leishmaniasis in the Muzaffapur Demographic Surveillance Site: A Spatiotemporal Analysis. Am J Trop Med Hyg 2018; 99(6):1555–61.

21. Stauch A, Duerr H-P, Picado A, Ostyn B, Sundar S, Rijal S et al. Model-based investigations of different vector-related intervention strategies to eliminate visceral leishmaniasis on the Indian subcontinent. PLoS Negl Trop Dis 2014; 8(4):e2810.
